# Methylome-wide analysis in systemic microbial-induced experimental periodontal disease in mice with different susceptibility

**DOI:** 10.3389/fcimb.2024.1369226

**Published:** 2024-07-16

**Authors:** Cristhiam de Jesus Hernandez Martinez, Joseph Glessner, Livia Sertori Finoti, Pedro Felix Silva, Michel Messora, Ricardo Della Coletta, Hakon Hakonarson, Daniela Bazan Palioto

**Affiliations:** ^1^ Department of Oral & Maxillofacial Surgery and Periodontology, Ribeirão Preto Dental School, University of São Paulo - USP, Ribeirão Preto, São Paulo, Brazil; ^2^ The Center for Applied Genomics, Children’s Hospital of Philadelphia, Philadelphia, PA, United States; ^3^ Department of Pediatrics, The Perelman School of Medicine, University of Pennsylvania, Philadelphia, PA, United States; ^4^ Division of Human Genetics, Children’s Hospital of Philadelphia, Philadelphia, PA, United States; ^5^ Laboratory of Rebecca Ahrens-Nicklas,Children’s Hospital of Philadelphia, Philadelphia, PA, United States; ^6^ Department of Oral Diagnosis and Graduate Program in Oral Biology, Piracicaba Dental School, University of Campinas, Piracicaba, Brazil; ^7^ Division of Pulmonary Medicine, Children’s Hospital of Philadelphia, Philadelphia, PA, United States; ^8^ Faculty of Medicine, University of Iceland, Reykjavik, Iceland

**Keywords:** periodontal disease, methylome, DNA methylation, mouse models, *Porphyromonas gingivalis*, differential methylome analysis

## Abstract

**Objective:**

The study delved into the epigenetic factors associated with periodontal disease in two lineages of mice, namely C57bl/6 and Balb/c. Its primary objective was to elucidate alterations in the methylome of mice with distinct genetic backgrounds following systemic microbial challenge, employing high-throughput DNA methylation analysis as the investigative tool.

**Methods:**

*Porphyromonas gingivalis* (*Pg*)was orally administered to induce periodontitis in both Balb/c and C57bl/6 lineage. After euthanasia, genomic DNA from both maxilla and blood were subjected to bisulfite conversion, PCR amplification and genome-wide DNA methylation analysis using the Ovation RRBS Methyl-Seq System coupled with the Illumina Infinium Mouse Methylation BeadChip.

**Results:**

Of particular significance was the distinct methylation profile observed within the *Pg*-induced group of the Balb/c lineage, contrasting with both the control and *Pg*-induced groups of the C57bl/6 lineage. Utilizing rigorous filtering criteria, we successfully identified a substantial number of differentially methylated regions (DMRs) across various tissues and comparison groups, shedding light on the prevailing hypermethylation in non-induced cohorts and hypomethylation in induced groups. The comparison between blood and maxilla samples underscored the unique methylation patterns specific to the jaw tissue. Our comprehensive methylome analysis further unveiled statistically significant disparities, particularly within promoter regions, in several comparison groups.

**Conclusion:**

The differential DNA methylation patterns observed between C57bl/6 and Balb/c mouse lines suggest that epigenetic factors contribute to the variations in disease susceptibility. The identified differentially methylated regions associated with immune regulation and inflammatory response provide potential targets for further investigation. These findings emphasize the importance of considering epigenetic mechanisms in the development and progression of periodontitis.

## Introduction

Periodontitis is one of the most prevalent chronic inflammatory conditions globally, primarily associated with a dysbiotic biofilm rather than specific pathogens (Darveau, 2010). This condition results in irreversible destruction of periodontal tissues, significantly impacting oral health (Hajishengallis, 2014a). Traditional views attributed periodontitis to specific bacteria like *Porphyromonas gingivalis, Tannerella forsythia*, and *Treponema denticola*. However, current understanding emphasizes a dysbiotic microbial community and a complex host immune response as central to disease progression (Darveau, 2010).

The dysbiotic progression triggers a cascade of events, including the release of cytokines, chemokines, and matrix-degrading enzymes, which exacerbate tissue destruction (Hajishengallis, 2014a). Elevated levels of inflammatory mediators such as prostaglandin E2 (PGE2), interleukin-1 (IL-1), tumor necrosis factor-α (TNF-α), IL-8, and interferon-γ (IFN-ɣ) are commonly found in diseased periodontal sites, leading to further inflammation and extracellular matrix breakdown (Bartold et al., 2010; Zhang et al., 2010b; Karimbux et al., 2012; Murray et al., 2020). Risk factors such as tobacco use and genetic predispositions, including specific single nucleotide polymorphisms (SNPs) in genes encoding inflammatory cytokines and receptors like TLR-2, TLR-4, CD-14, and COX-2, also contribute to susceptibility (Chen et al., 2006; Nibali et al., 2007; Nibali et al., 2008).

Epigenetic modifications, including DNA methylation and histone modifications, play a crucial role in gene expression regulation without altering the DNA sequence itself. These modifications have been extensively studied in various diseases, including periodontitis, highlighting their role in the disease’s pathogenesis (Bartold et al., 2010). DNA methylation, which typically represses gene transcription, has been implicated in regulating inflammatory responses in periodontitis. Altered methylation patterns in periodontal tissues have been observed, particularly in genes related to inflammation and immune response (Zhang et al., 2010a). For instance, hypermethylation of the IL-10 gene promoter region is associated with decreased expression of this anti-inflammatory cytokine, contributing to chronic inflammation in periodontitis (Bartold et al., 2010).

Histone modifications, such as acetylation, methylation, phosphorylation, and ubiquitination, also play a vital role in chromatin structure and gene expression regulation. These modifications influence the expression of genes involved in immune and inflammatory responses in periodontitis. For example, acetylation of histones H3 and H4 at pro-inflammatory cytokine gene promoters like TNF-α and IL-6 correlates with increased expression in periodontal lesions (Reyes et al., 2013).

The interplay between DNA methylation and histone modifications further complicates the epigenetic regulation in periodontitis. These modifications can act synergistically or antagonistically to regulate gene expression, leading to the aberrant expression of genes involved in inflammation, immune response, and tissue remodeling, thereby contributing to periodontitis pathogenesis (McCawley and Matrisian, 2000). Understanding the role of epigenetic modifications in periodontitis provides insights into the disease’s molecular mechanisms and opens new avenues for therapeutic strategies. Targeting specific epigenetic modifications may offer novel approaches to modulate gene expression and inflammatory responses in periodontal disease.

Although studies on epigenetics and periodontal disease are still in their early stages, accumulating evidence over the past decade has shown the involvement of epigenetic modifications in periodontitis, explaining the silencing or overexpression of certain genes related to different disease stages (Bartold et al., 2010). The modulation of various immune system components, regulated by genetic and epigenetic mechanisms, plays a crucial role in the development of susceptibility to periodontal disease (Hajishengallis, 2014a; Hajishengallis, 2014b; Hajishengallis and Lamont, 2014).

Research into genetics, epigenetics, host environmental exposures, and animal models of periodontitis has provided significant insights. Studies have demonstrated that induced bone loss in periodontitis depends on the host’s genetic background, epigenetic factors, and oral microbiome composition (Reyes et al., 2013). Cross-breeding studies have shown that the inheritance of disease outcome is closely tied to genetic factors. Variations in alveolar bone loss can be observed in different mouse lineages, suggesting that immune response differences, such as specific mutations in Toll-like receptor 4 (TLR4), contribute to these variances (Chen et al., 2006).

The study of DNA methylation profiles in periodontitis has proven crucial, as illustrated through diverse models. For example, in a C57BL/6 mouse model of periodontitis, researchers observed increased bone loss associated with epigenetic-inflammatory changes, notably the overexpression of DNMT3b following systemic exposure to *Porphyromonas gingivalis* (Palioto et al., 2019). Similarly, the inhibition of DNMT1 with 5-Aza-cytidine in fibroblasts treated with TGF-β1 (from either gingival or periodontal ligament) resulted in decreased DNA methylation and an upregulation of IL-11 expression (Sufaru et al., 2017). These findings underscore the role of epigenetic mechanisms, like DNA methylation, in modulating gene expression that influences the host’s response to periodontal pathogens.

Subsequent research, including studies by Tanaka U et al. (2021) (Tanaka et al., 2021) and Kin H et al. (2021) (Kim et al., 2021), has delved into the methylation patterns linked to both periodontal health and disease. These studies have proposed that certain methylation changes could serve as valuable biomarkers for assessing disease severity and may also provide viable targets for therapeutic intervention. Furthermore, Benakanakere M et al. (2015) (Benakanakere et al., 2015) investigated the interplay between epigenetic alterations and the microbial landscape within the periodontal environment, emphasizing how variations in DNA methylation could significantly impact the course and intensity of periodontal disease. These insights underscore the critical role of epigenetic modifications in influencing disease dynamics and stress the need for targeted research to mitigate periodontal deterioration.

Moreover, recent studies by Lagosz-Cwik KB et al. (2023) (Lagosz-Cwik et al., 2023) and Cho Y et al. (2017) (Cho et al., 2017) have provided insights into the potential of epigenetic therapy in periodontal treatment. These investigations focused on the effects of reversing specific methylation patterns, opening new avenues for managing inflammation and tissue repair in periodontitis. Notably, Cho Y et al. (2017) (Cho et al., 2017) discussed how periodontal therapy, in relation to epigenetic changes, might not only halt disease progression but also potentially reverse harmful epigenetic modifications.

Collectively, these studies offer a compelling narrative that positions DNA methylation not merely as a marker of disease state but as a significant lever for therapeutic intervention, emphasizing the importance of continued research in this area to fully harness the therapeutic potentials of epigenetics in periodontal disease. This multifaceted approach highlights how a robust methodology and detailed assessment of DNA methylation levels could advance our understanding of periodontal health and individual variability in disease response.

## Materials and methods

### Ethical considerations

The experimental procedures conducted in this study received ethical approval from the Ethics Committee on Animal Use (CEUA) at the Ribeirão Preto School of Dentistry (FORP), University of São Paulo (USP), under protocol number CEUA 2018.1.644.58.6. Our research team took every possible measure to minimize animal suffering and reduce the number of animals used, in full compliance with the guidelines set forth by the Brazilian Society for Laboratory Animal Science (SBCAL/COBEA), which adhere to the ethical principles of animal experimentation. Our study also aligns with the regulations for the didactic-scientific practice of animal vivisection (Law 11.794/2008), the Universal Declaration of Animal Rights established by UNESCO (United Nations Educational, Scientific and Cultural Organization), and international standards outlined in the Guide for the Care and Use of Laboratory Animals by the National Academy of Sciences, USA (1996).

### Study design

The study involved a cohort of 12 male mice (Mus musculus), including 6 Balb/c mice and 6 C57bl/6 mice. To initiate the experimental protocol, the mice were subjected to a 10-day systemic administration of trimethoprim-sulfamethoxazole, at a dosage of 80 mg trimethoprim and 400 mg sulfamethoxazole in 5 ml deionized water. Each animal received sulfamethoxazole (0.87 mg/mL) and trimethoprim (0.17 mg/mL) once daily, according to established protocols in the literature (Nahid et al., 2011; Rivera et al., 2013; Shi et al., 2017; Palioto et al., 2019; Hernández Martínez C de et al., 2023) Following this treatment, a 3-day antibiotic-free period was implemented to reduce the native microbiota and ensure accurate experimental conditions.

### Preparation of *P. gingivalis* bacterial culture

The original *Pg* W83 strain was maintained in a frozen state at -80°C, stored in defibrinated sheep blood. It was sourced from the Microbiology Laboratory of the Department of Clinical, Toxicological, and Food Analysis at the Faculty of Pharmaceutical Sciences of Ribeirão Preto, University of São Paulo. For preservation, the bacteria underwent weekly transfers to blood agar plates supplemented with tryptic soy agar, 0.1% yeast extract, 5.0 μg/ml hemin, 0.5 μg/ml menadione, and 5% defibrinated sheep blood. In preparation for experiments, the bacteria were cultured under anaerobic conditions with an atmosphere of 5% CO2, 10% H2, and 85% N2 at 37°C for 4 to 7 days on the same supplemented blood agar. Subsequently, the bacteria were suspended in phosphate-buffered saline (PBS), and the number of colony-forming units (CFU) was standardized by measuring the optical density at 700 nm (Hernández Martínez C de et al., 2023).

### Systemic microbial challenge

**Figure 1:**
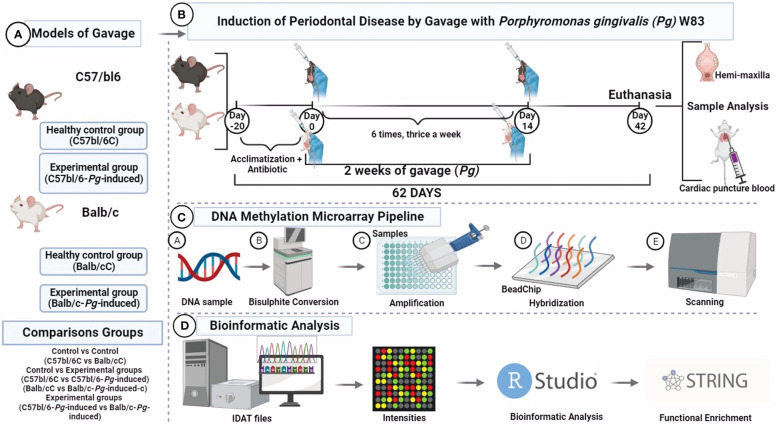
DNA methylone profiling. **(A)** Study groups; **(B)** Experimental design; **(C)** Mouse methylation BeadChip; **(D)** Bioinformatic analysis. Visualization created with BioRender.

Half of the mice of each group received *Pg* inoculation (experimental group) following the methods previously described elsewhere (Kato et al., 2018), whereas the other half was the control group. The study comprised two experimental groups: C57bl/6-*Pg*-induced and Balb/c-*P*g-induced. Correspondingly, the control groups consisted of C57bl/6C and Balb/cC lineages. In essence, a total of 10^9^ live colony forming units of *Pg* suspended in 200 µl of phosphate buffered saline (PBS) containing 2% sterile carboxymethyl cellulose were delivered via a feeding needle. This inoculation process was repeated three times with 2-day intervals per week, spanning a total duration of 2 weeks. The euthanasia occurred precisely 62 days after the initiation of the experimental procedures. Intraperitoneal administration of a lethal dose of pentobarbital at 150 mg/kg was utilized for the euthanasia process, ensuring a humane and effective method of termination (**Figure 1**).

### Sample collection and processing

Bone (maxilla) and blood obtained by cardiac puncture were collected during necropsy. The bone was cryopreserved in liquid nitrogen, and the blood was stored in sterile tubes containing ethylenediaminetetraacetic acid (EDTA) and stored at -80°C.

#### Bone samples

Bone samples were collected post-necropsy and immediately cryopreserved in liquid nitrogen at -125°C. This ultra-low temperature storage is essential for preserving nucleic acids by minimizing enzymatic activities and preventing DNA degradation. The cryopreserved bone samples were first brought to liquid nitrogen temperature before being pulverized into a fine powder using a mortar and pestle under liquid nitrogen, ensuring that the samples remained frozen to prevent thawing and potential DNA degradation (Kalendar et al., 2021).

#### Blood samples

Blood samples were collected via cardiac puncture during necropsy and placed in sterile tubes containing ethylenediaminetetraacetic acid (EDTA). EDTA acts as an anticoagulant and chelating agent, inhibiting nucleases that could degrade DNA. The blood samples were then stored at -80°C to further preserve the DNA integrity. The combination of EDTA and low-temperature storage is critical for maintaining the stability of the nucleic acids in the blood samples (Gupta, 2019).

#### DNA isolation from bone

The process begins with approximately 100 mg of the pulverized bone powder being transferred to a microcentrifuge tube containing 1 ml of lysis buffer (10 mM Tris-HCl, 100 mM NaCl, 25 mM EDTA, 0.5% SDS). Proteinase K was added to the mixture to a final concentration of 0.1 mg/ml, and the sample was incubated at 56°C overnight to ensure complete lysis of cells and digestion of proteins. The lysate was then subjected to phenol-chloroform extraction to remove proteins and other contaminants. Equal volumes of phenol and chloroform were added to the lysate, mixed thoroughly, and centrifuged at 12,000 g for 10 minutes. The aqueous phase, containing the DNA, was carefully transferred to a new tube. DNA was precipitated by adding 2.5 volumes of cold ethanol and 0.1 volume of 3 M sodium acetate (pH 5.2) to the aqueous phase. The mixture was incubated at -20°C for at least 1 hour and then centrifuged at 12,000 g for 15 minutes to pellet the DNA. The DNA pellet was washed with 70% ethanol, air-dried, and dissolved in TE buffer (10 mM Tris-HCl, 1 mM EDTA, pH 8.0) (Kalendar et al., 2021).

#### DNA isolation from blood

EDTA-treated blood samples stored at -80°C were thawed on ice. A 500 µl aliquot of the blood sample was mixed with 1 ml of red blood cell lysis buffer (155 mM NH4Cl, 10 mM KHCO3, 0.1 mM EDTA) and incubated on ice for 10 minutes to lyse the red blood cells. The sample was centrifuged at 300 g for 5 minutes, and the supernatant was discarded. The pellet, containing white blood cells, was resuspended in 500 µl of white blood cell lysis buffer (10 mM Tris-HCl, 400 mM NaCl, 2 mM EDTA, 0.2% SDS). Proteinase K was added to a final concentration of 0.1 mg/ml, and the mixture was incubated at 56°C for 1-2 hours. Similar to the bone DNA extraction, phenol-chloroform extraction was performed to purify the DNA. Equal volumes of phenol and chloroform were added, mixed, and centrifuged at 12,000 g for 10 minutes. The aqueous phase was transferred to a new tube. DNA was precipitated by adding 2.5 volumes of cold ethanol and 0.1 volume of 3 M sodium acetate (pH 5.2), followed by incubation at -20°C for 1 hour and centrifugation at 12,000 g for 15 minutes. The DNA pellet was washed with 70% ethanol, air-dried, and dissolved in TE buffer (Shams et al., 2011; Kalendar et al., 2021).

### Quality control

The quality and quantity of the isolated DNA were assessed using spectrophotometry, with the A260/A280 ratio indicating purity (ideal range: 1.8-2.0). Agarose gel electrophoresis was used to check for intact high-molecular-weight DNA and confirm the absence of degradation. Ensuring high purity and integrity of the isolated DNA is essential for obtaining accurate and reliable data in methylation array analysis.

The detailed bioinformatics analysis is supplied in the **Supplementary Materials** and **Supplementary Methods**.

## Results

After a meticulous categorization based on tissue type and experimental grouping, avoiding potential cross-contaminations that could compromise the fidelity of the results, global DNA methylation profiles were compared between mouse strains. Of the 286,212 comprehensive probes analyzed, a substantial proportion showed no discernible changes. However, upon applying the filtering criterion (delta beta methylation difference of ≥ 20%), notable disparities emerged across all comparison cohorts, as elucidated in **Figures 2.1** and **2.2**.

**Figure 2:**
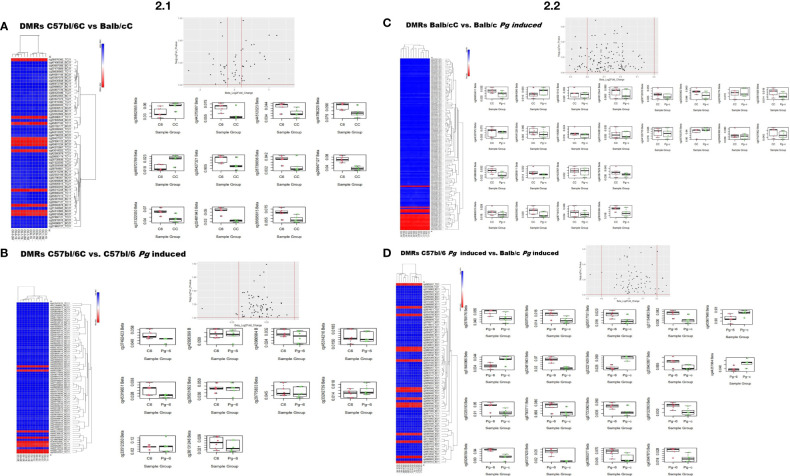
Methylation profiling of Periodontal disease case samples versus control samples. 2.1 **(A)** Differentially methylated probes (DMRs) of the control groups C57bl/6 and Balb/c (C57bl/6C vs Balb/cC); **(B)** Differentially methylated probes (DMRs) of the control group versus gavage C57bl/6 (C57bl/6C vs C57bl/6-*Pg*-induced); 2.2 **(C)** Differentially methylated probes (DMRs) of the control group versus gavage Balb/c (Balb/cC vs Balb/c-*Pg*-induced); **(D)** Differentially methylated probes (DMRs) from C57bl/6 versus Balb/c gavage groups (C57bl/6-*Pg*-induced vs Balb/c-*Pg*-induced).

The heatmap illustrates differential methylation in various loci between the C57bl/6C and Balb/cC groups (**Figure 2.1A**). Some loci show significant differential methylation between the groups, with red regions indicating highly methylated loci and blue regions indicating low methylation. The dendrogram reveals hierarchical clustering, indicating that certain loci group according to their methylation levels, suggesting specific methylation patterns in each group. Regions such as cg36540786, cg46160718, and cg26060778 exhibit distinct methylation patterns between the groups, indicating important differentially methylated regions (DMRs). The scatter plot shows most points concentrated around the vertical axis (log2 fold change near zero), with some dispersions in both directions, suggesting that many of the observed methylation changes are small. Some points above the horizontal line indicate statistically significant differentially methylated regions. Points to the right of the right vertical line and above the horizontal line indicate regions that are significantly more methylated in C57bl/6C, while points to the left of the left vertical line and above the horizontal line indicate regions that are significantly more methylated in Balb/cC (**Figure 2.1A**).

Notable differences in methylation levels are observed between the groups for several loci. CpG sites such as cg3655250, cg4476225, cg4824721, and cg4924717 show hypermethylation in the C57bl/6C group compared to the Balb/cC group, whereas CpG sites such as cg4437507, cg4677208, cg3132200, and cg5505615 show hypomethylation in the C57bl/6C group compared to the Balb/cC group. Some CpG sites, such as cg4451202, cg4873968, and cg3248194, show no clear difference between the C57bl/6C and Balb/cC groups (**Figure 2.1A**).

The heatmap shows that the samples tend to cluster according to their conditions (C57bl/6C vs C57bl/6C-*Pg*-induced) (**Figure 2.1B**), suggesting that there are consistent methylation patterns that differentiate the two groups. Some CpG sites, such as those in the middle and near the end of the graph, show distinct methylation patterns between the groups, indicating important DMRs. The dendrogram suggests that certain loci are differentially methylated between the groups, indicating possible epigenetic changes induced by *Pg* treatment. The scatter plot shows most points concentrated around the vertical axis (log2 fold change near zero), with some dispersions in both directions, suggesting that many of the observed methylation changes are small. Points to the right of the right vertical line and above the horizontal line indicate regions that are significantly more methylated in C57bl/6-*Pg*-induced, while points to the left of the left vertical line and above the horizontal line indicate regions that are significantly more methylated in C57bl/6C. For all the shown CpG sites, beta values are consistently higher in the C57bl/6-*Pg*-induced group compared to the C57bl/6C group, indicating hypermethylation in C57bl/6-*Pg*-induced. For example, loci such as cg09270590, cg20571082, and cg22701270 show clear methylation differences between C57bl/6C (in red) and C57bl/6-*Pg*-induced (in green), suggesting differential epigenetic regulation in response to *Pg* treatment. This pattern suggests that induction by C57bl/6-*Pg*-induced leads to a general increase in methylation levels across various CpG sites (**Figure 2.1B**).

In **Figure 2.2C**, the heatmap shows that most loci exhibit low methylation (indicated by blue regions), but some loci show high methylation (indicated by red regions), primarily in the Balb/c-*Pg*-induced group. The dendrogram indicates hierarchical clustering, suggesting specific methylation patterns for each group. The samples tend to cluster according to their conditions (Balb/cC vs Balb/c-*Pg*-induced), suggesting consistent methylation patterns that differentiate the two groups. Some CpG sites, such as those at the bottom of the graph, show distinct methylation patterns between the groups, indicating important DMRs. The scatter plot shows points above the horizontal line that indicate statistically significant differentially methylated regions. These loci are considered differentially methylated, indicating possible regulatory regions affected by *Pg* induction. Points to the right of the right vertical line and above the horizontal line indicate regions that are significantly more methylated in Balb/c-*Pg*-induced, while points to the left of the left vertical line and above the horizontal line indicate regions that are significantly more methylated in Balb/cC. Significant differences in methylation levels are observed between the groups for several loci. For example, loci such as cg02121310, cg05417851, and cg09957274 show clear methylation differences between Balb/cC (in red) and Balb/c-*Pg*-induced (in green). Of the 24 probes analyzed, 16 showed significant CpG sites, with 11 being hypomethylated and 5 hypermethylated, confirming a significant trend of hypomethylation in the *Pg*-induced group.

**Figure 2.2D** shows that several loci exhibit distinct methylation patterns between the C57bl/6-*Pg*-induced and Balb/c-*Pg*-induced groups. Regions consistently red or blue in one group but not the other indicate significant methylation differences. Some CpG sites, such as those in the middle and near the end of the graph, show distinct methylation patterns between the groups, indicating important DMRs. The hierarchical clustering suggests that certain loci behave similarly in terms of methylation, implying co-regulation or shared epigenetic effects between these loci. The scatter plot shows points above the horizontal line and outside the vertical lines as of greatest interest, as they represent statistically significant DMRs with substantial methylation changes. Most points are concentrated around the vertical axis (log2 fold change near zero), with some dispersions in both directions, suggesting that many of the observed methylation changes are small. Points to the right of the right vertical line and above the horizontal line indicate regions that are significantly more methylated in C57bl/6-*Pg*-induced, while points to the left of the left vertical line and above the horizontal line indicate regions that are significantly more methylated in Balb/c-*Pg*-induced.

Significant differences in methylation levels are observed between the groups for several loci. Examples of loci with notable differences include cg07058781, where the Balb/c-*Pg*-induced group shows higher methylation levels compared to the C57bl/6-*Pg*-induced group, indicating possible epigenetic regulation specific to the Balb/c-*Pg*-induced group. The loci cg09781789 shows lower methylation levels in the Balb/c-*Pg*-induced group, suggesting specific hypomethylation in this group. The variation in methylation levels at loci cg15081391 is greater in the C57bl/6-*Pg*-induced group, indicating a more heterogeneous epigenetic response to *Pg* treatment (**Figure 2.2D**). Additionally, loci such as cg22858999 show higher methylation in the Balb/c-*Pg*-induced group, suggesting possible activation or repression of specific genes due to the treatment. At loci like cg23661285, methylation is significantly higher in the C57bl/6-*Pg*-induced group, suggesting a specific response to the gavage group.

The differences in DNA methylation observed between the C57bl/6-*Pg*-induced and Balb/c-*Pg*-induced groups suggest that *Pg* treatment has specific epigenetic effects on the two mouse strains, reflecting variations in gene expression responsible for specific phenotypic responses. These results highlight the importance of epigenetic modifications in response to *Pg* challenge in different mouse strains, providing a solid foundation for future investigations into epigenetic regulation and its impact on mouse biology, potentially contributing to the development of therapeutic interventions based on epigenetic modifications.

To explore potential DNA methylation markers associated with periodontal disease risk, regardless of *Pg* status, we conducted a comprehensive methylome analysis. Evaluating transcription start sites (TSS) (**Figure 3.1**), it is observed that hypermethylated regions are more numerous than hypomethylated regions, suggesting that hypermethylation is a common and dominant phenomenon in the comparisons made. Regions larger than 100 kb are the most common among hypermethylated regions, indicating that methylation tends to affect large genome segments. *Pg* induction appears to increase the number of hypermethylated regions but also affects hypomethylated regions, though to a lesser extent.

**Figure 3:**
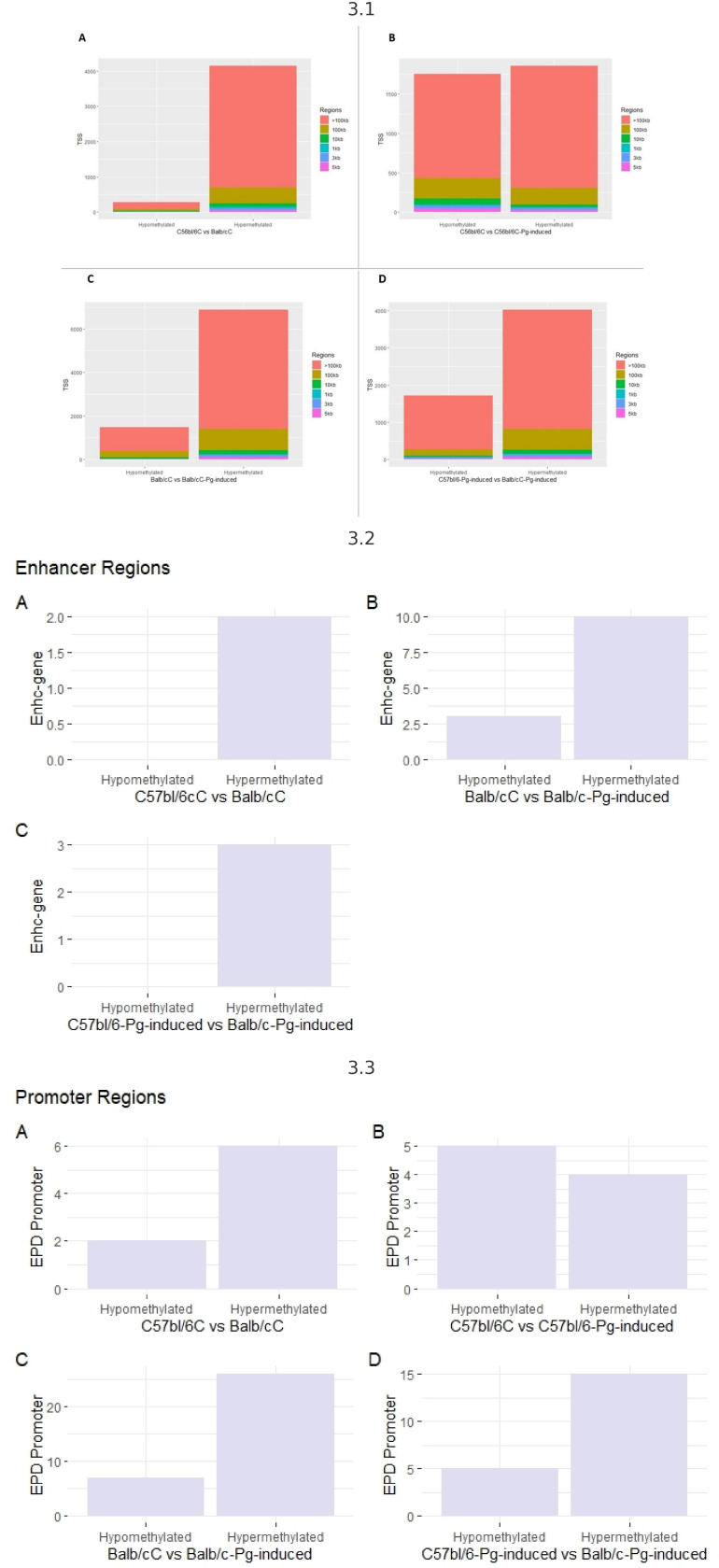
3.1 - TSS transcription start sites. **(A)** Control groups (C57bl/6C vs Balb/cC); **(B)** Control group versus gavage (C57bl/6C vs C57bl/6-*Pg*-induced); **(C)** Control group versus gavage Balb/c (Balb/cC vs Balb/c-*Pg*-induced); **(D)** Gavage groups C57bl/6 versus Balb/c (C57bl/6-*Pg*-induced vs Balb/c-*Pg*-induced). 3.2- Enhancer (potentiator) regions. **(A)** Control groups (C57bl/6C vs Balb/cC); **(B)** Control group versus gavage Balb/c (Balb/cC vs Balb/c-*Pg*-induced); **(C)** Gavage groups C57bl/6 versus Balb/c (C57bl/6 -*Pg*-induced vs Balb/c-*Pg*-induced). 3.3 - Promoter regions. **(A)** Control groups (C57bl/6C vs Balb/cC); **(B)** Control group versus gavage C57bl/6 (C57bl/6C vs C57bl/6-*Pg*-induced); **(C)** Control group versus gavage Balb/c (Balb/cC vs Balb/c-*Pg*-induced); **(D)** Gavage groups C57bl/6 versus Balb/c (C57bl/6-*Pg*-induced vs Balb/c-*Pg*-induced).

Hypermethylation of enhancer regions (**Figure 3.2**) is a predominant phenomenon, especially after *Pg* induction. In graph B, *Pg* induction in Balb/C significantly increases the number of hypermethylated enhancer regions. Comparing C57bl/6-*Pg*-induced with Balb/C-*Pg*-induced (Graph C), we see a predominance of hypermethylated enhancer regions, with no hypomethylated regions due to the absence of modifications for the C57bl/6C vs C57bl/6-*Pg*-induced comparison. In graphs A and C, there are no records of hypomethylated enhancer regions, indicating that methylation in enhancer regions tends to be more frequently increased (hypermethylated) rather than reduced (hypomethylated) in these contexts. *Pg* induction significantly increases the number of hypermethylated enhancer regions in Balb/C.

In “EPD Promoter” regions, in all comparisons, hypermethylated promoter regions are more numerous than hypomethylated regions, except in graph B where there is a greater balance between hypomethylation and hypermethylation (**Figure 3.3**). *Pg* induction in Balb/C (Graph C) significantly increases the number of hypermethylated promoter regions. Comparing C57bl/6-*Pg*-induced with Balb/C-*Pg*-induced (Graph D), there is a significant predominance of hypermethylated promoter regions. In graph B, the distribution of hypomethylated and hypermethylated promoter regions in C57bl/6 vs C57bl/6-*Pg*-induced is almost balanced, suggesting that *Pg* induction does not cause a drastic change in promoter region methylation in C57bl/6. *Pg* induction tends to significantly increase the number of hypermethylated promoter regions, especially in Balb/C.

**Figure 4:**
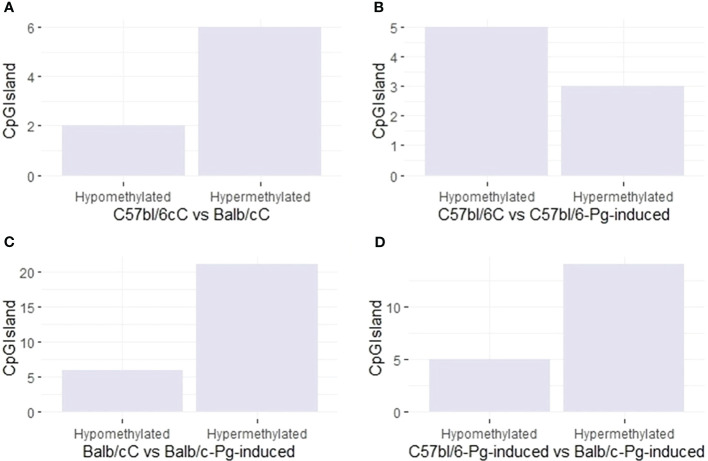
CpG site types. **(A)** Control groups (C57bl/6C vs Balb/cC); **(B)** Control group versus gavage C57bl/6 (C57bl/6C vs C57bl/6-*Pg-*induced); **(C)** Control group versus gavage Balb/c (Balb/cC vs Balb/c-*Pg*-induced); **(D)** Gavage groups C57bl/6 versus Balb/c (C57bl/6-*Pg-*induced vs Balb/c-*Pg*-induced).

*Pg* induction has a variable effect on the methylation of CpGIsland regions (**Figure 4**). In graphs A and C, hypermethylation is more common than hypomethylation, especially in Balb/C and after *Pg* induction. In C57bl/6 (Graph B), *Pg* induction results in more hypomethylation than hypermethylation. In Balb/C (Graph C), *Pg* induction results in a significant increase in hypermethylation. In graph D, comparing C57bl/6-*Pg*-induced with Balb/C-*Pg*-induced, there is an almost equal balance between hypomethylation and hypermethylation, with a slight predominance of hypermethylation. This may suggest that *Pg* induction causes methylation changes that are approximately balanced between the two groups, with hypermethylation of CpGIsland regions being more common in comparisons involving Balb/C and after *Pg* induction.

Methylation levels in different blood and jaw samples are shown in **Figure 5.1**, with colors ranging from blue (minimum level) to red (maximum level). The columns represent different samples, and the rows represent different genes or CpG regions. The dendrogram above the heatmap indicates sample clusters based on the similarity of expression or methylation profiles. It was evident that jaw samples showed a higher number of differentially methylated genes, as indicated by the expanded branch in the hierarchical clustering.

**Figure 5:**
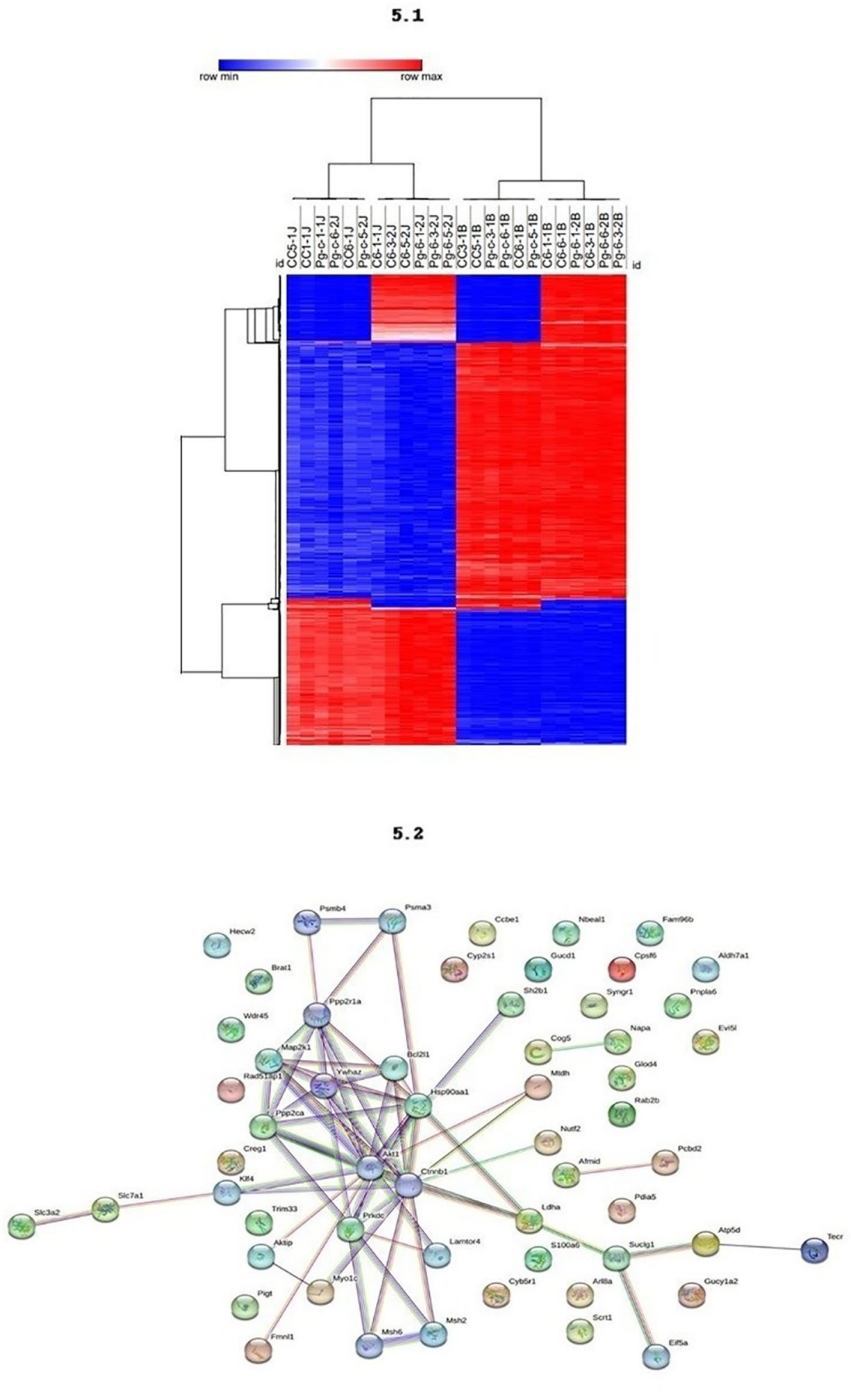
5.1 - The methylation profiling of the DMRs across different tissues. DNA methylation levels in blood and maxilla of each DMR across two tissues. The colors in the heatmap indicate the high (red) or low (blue) DNA methylation levels. 5.2 - Functional Enrichment. Protein-protein interaction network with the genes associates to periodontal disease.

The PPI network analysis reveals central proteins, such as Akt1, Ctnnb1, and Hsp90aa1, that play crucial roles in regulating key cellular processes. The identification of functional clusters indicates specific molecular pathways that may be differentially regulated between the study groups (**Figure 5.2**). Akt1, identified as a highly connected central node, is involved in several cellular signaling pathways, including the regulation of cell growth, survival, and metabolism. Its numerous connections suggest that Akt1 may be a master regulator in the network. Ctnnb1 (Beta-catenin), another central node, crucial in the Wnt signaling pathway, regulates gene expression and cell adhesion, indicating its importance in maintaining cellular integrity and signal transduction. Hsp90aa1, a heat shock protein essential for protein folding and stability, has multiple connections suggesting a vital role in cellular stress response and protein homeostasis. The densely connected interactions indicate a highly integrated functional network, possibly responsible for coordinated responses to external stimuli.

Specific subclusters, such as Slc7a1 and Slc3a2, strongly connected, indicate a specific functional interaction, possibly related to amino acid transport. Another subcluster, consisting of Cog5, Mtdh, Glod4, and Rab2b, suggests a common function, potentially in vesicular trafficking and regulation of cellular metabolism.

## Discussion

Building upon our prior investigation into the chronology of alveolar bone loss following systemic exposure to *Porphyromonas gingivalis* W83, marked dissimilarities have come to light (Hernández Martínez C de et al., 2023). This prompted a meticulous examination of methylation patterns within these two strains at the two-week juncture, with the overarching goal of unraveling the root causes behind the observed epigenetic variations and shedding light on potential explanations for their distinct susceptibilities. Our study has yielded compelling insights into the divergent methylation patterns exhibited by the C57bl/6 and Balb/c mouse lineages, renowned for their disparate susceptibility to periodontal disease, C57bl/6 mice are characterized by a Th1-biased immune response, producing higher levels of pro-inflammatory cytokines such as IFN-γ, which is essential for cell-mediated immunity and the control of intracellular pathogens, often resulting in a more robust inflammatory response (Jankovic et al., 2001; Watanabe et al., 2004). Conversely, Balb/c mice exhibit a Th2-biased immune response, with elevated levels of cytokines such as IL-4, IL-5, and IL-13, which are associated with humoral immunity and B cell activation. This Th2 dominance can result in a more effective response against extracellular pathogens but might render these mice more susceptible to chronic inflammatory conditions due to a less controlled pro-inflammatory response (Jankovic et al., 2001; Watanabe et al., 2004).

Research suggests that C57bl/6 mice may demonstrate a more controlled immune response in the context of periodontal disease, potentially limiting the extent of periodontal tissue destruction. In contrast, Balb/c mice might experience more severe periodontal disease due to a less regulated inflammatory response, resulting in greater tissue damage and susceptibility to infection (Baker et al., 1994; Baker et al., 2000; Sasaki et al., 2004; Baker, 2005; Graves et al., 2008). Comparative studies of these strains in microbial challenges consistently highlight these differences, with C57bl/6 mice often showing a higher capacity for bacterial clearance and a more aggressive initial immune response, which is beneficial for preventing chronic infection but may also result in more pronounced acute inflammation (Watanabe et al., 2004). Balb/c mice, however, may exhibit a more subdued initial response, allowing for greater bacterial colonization and chronicity of infection, thus providing a contrasting model for studying the long-term effects of microbial challenges and resultant methylome alterations (Jankovic et al., 2001). As demonstrated in our study, the Balb/c strain developed greater bone loss compared to the C57bl/6 strain (Hernández Martínez C de et al., 2023).

The Balb/cC and Balb/c-*Pg*-induced group exhibited a higher number of hypomethylated regions compared to the control groups (C57bl/6C) and the C57bl/6-*Pg*-induced-group, potentially influenced by specific genetic and epigenetic backgrounds (Dawson). Hypermethylation in control groups suggests the suppression of specific genes, which, when their suppression is removed by demethylation, may promote periodontitis. Similarly, hypomethylation-rich pattern in control groups may indicate the protective role of specific genes in preventing periodontitis. The concept of “tumor suppressor genes” and “oncogenes” in cancer can be paralleled with our findings, where the hypermethylated genes in control groups are predominantly oncogenes, while the hypomethylated genes are predominantly tumor suppressor genes. Balb/c mice are known to have deficiencies in the innate immune system, including impaired neutrophil function and reduced production of pro-inflammatory cytokines (Dutzan et al., 2017). These immune deficiencies, combined with DNA hypomethylation, may contribute to increased susceptibility to periodontal disease by affecting key disease-related pathways. Therefore, understanding the interplay between genetic and epigenetic factors, along with immune responses, provides insights into the mechanisms underlying periodontal disease susceptibility and potential therapeutic targe (Dawson; Li et al., 2015; Dutzan et al., 2017).

In agreement with previous findings (Dawson), our results indicate that the hypomethylated regions may affect the regulation of extracellular matrix remodeling and tissue homeostasis by influencing the expression of matrix metalloproteinases (MMPs) and tissue inhibitors of metalloproteinases (TIMPs). Balb/c mice have been shown to exhibit hypomethylation in the promoter regions of genes encoding MMPs, potentially leading to increased matrix degradation and tissue destruction in response to periodontal pathogens (Dawson). Interestingly, we observed differences in the epigenetic regulation of enhancer regions between the Balb/c and C57bl/6 lineages. Genetic variation between the lineages can influence the expression and activity of enhancers, which play a crucial role in regulating gene expression and immune responses (Li et al., 2020). Epigenetic modifications, such as DNA methylation, histone modifications, and chromatin accessibility, can also modulate enhancer activity (Wilson, 2008). Balb/c and C57bl/6 mice exhibit differences in DNA methylation patterns and histone modifications (Schmidt et al., 2017), which may contribute to the observed disparities in enhancer regulation. These differences in enhancer regulation may be linked to variations in immune responses and inflammation, as enhancers control immune-related genes and modulate immune cell function and inflammatory processes (Heinz et al., 2015; Li et al., 2015; Dutzan et al., 2017). Notably, the absence of results in the C57bl/6 group regarding enhancers and silencers (cis-regulatory elements) does not definitively establish that this lineage is more resistant to periodontal disease, but it may indicate that this difference with Balb/c exists because the critical “onset/activation” time of the chronic condition has passed, influenced by multiple factors, including genetics, epigenetics, environment and microbiome. These factors contribute to increased chromatin accessibility towards transcriptional events. Consequently, a partial acquisition of markers such as histone acetylation or methylation takes place, which may not be epigenetically marked in unstimulated cells. However, in cells undergoing typical active enhancer modifications (e.g., H3K4me1 - monomethylation of histone H3 at K4), the concept of trained immunity emerges (Ostuni et al., 2013; Hajishengallis et al., 2019; Li et al., 2022).

Built upon the framework of trained immunity (Hajishengallis et al., 2019), the present study proposes that distinct animal lineages possess differing epigenetic backgrounds. These variations lead to a diversified activation of the immune system, resulting in tissue protection against systemic *Pg* challenges, as well as variable responses to bone loss. These findings are grounded in the notion that epigenetic modifications also underlie the phenomenon of myeloid cells, such as macrophages, executing tissue-specific functions, heavily guided by microenvironmental stimuli (Matzinger, 2007; Stout et al., 2009). Indeed, the local microenvironment shapes the macrophage enhancer landscape beyond developmental origin attributions, thus contributing to their plasticity within specific tissue contexts (Lavin et al., 2014; Hajishengallis et al., 2019; Li et al., 2022).

CpG islands are regulatory regions located near gene promoters, and their methylation status can influence gene expression (Li et al., 2015; Li et al., 2022). Studies have shown that promoter methylation is crucial in regulating gene expression and phenotypic variation (Zhang et al., 2020; de Mendoza et al., 2022). The investigation by Sartori-Valinotti et al. (2007) (Sartori-Valinotti et al., 2007) also revealed dynamic changes in DNA methylation in response to bacterial infection in a mouse model, affecting immune response-related genes.

The results of this study revealed that CpG sites within differentially methylated regions (DMR) were enriched in the promoter regions, supporting the importance of promoter methylation in regulating gene expression and influencing phenotypic variation (Jones et al., 2016). Additionally, the enrichment patterns of hypomethylated and hypermethylated probes in the C57bl-6 vs Pg-induced-6 group were slightly similar in the promoter regions, suggesting that both hypomethylation and hypermethylation may contribute to differential gene regulation in this specific comparison group.

Moreover, our results demonstrated significant enrichment of hypermethylated probes in CpG island regions for both Balb/cC vs Balb/c-Pg-induced and C57bl/6-Pg-induced vs Balb/c-Pg-induced comparisons. These findings provide insights into the differential methylation patterns in response to *Pg W83* infection in blood and maxilla samples of C57bl/6 and Balb/c mouse lineages. The greater number of differentially methylated genes in the blood samples suggests a systemic impact of the infection on DNA methylation profiles, while the localized differences in the maxilla samples reflect the specific tissue response to the periodontal pathogen.

These findings support the notion that infections can elicit systemic alterations in DNA methylation profiles. Furthermore, the greater extent of differential methylation in the blood samples may reflect the involvement of immune-related pathways and processes. Immune cells in the bloodstream play a crucial role in detecting and responding to pathogens, and alterations in DNA methylation patterns can influence immune cell function and the overall immune response. This is consistent with the study by Li et al. (2020) (Li et al., 2020), which reported immune-related differentially methylated genes in peripheral blood cells in response to infection. In contrast, the observed differences in methylation patterns in the maxilla samples may be more localized and specific to the affected tissues. The maxilla is a key anatomical site for the progression of periodontal disease, and *Pg* W83 is a known periodontal pathogen. Studies have shown that DNA methylation changes in the maxilla can influence the expression of genes associated with periodontal inflammation and tissue destruction. For instance, Nakajima et al. (2017) (Kubo et al., 2015) demonstrated differential methylation patterns in the maxilla associated with immune response and bone remodeling during periodontitis progression. Influential research by Sorsa et al. (2016) (Sorsa et al., 2016) and Paul O et al. (2021) (Paul et al., 2021) underscores that inflammatory markers found in blood samples reflect the systemic nature of periodontal disease, thereby offering a comprehensive assessment of its impact on overall health (MaChado et al., 2021). Furthermore, the examination of the maxilla provides indispensable data on local structural and molecular alterations, contributing significantly to understanding disease progression, as highlighted by the studies of Oz et al. (2010) (Oz and Ebersole, 2010; Oz et al., 2010).

While gingival tissue visibly manifests the inflammatory processes typical of periodontal disease, the integration of blood sample analyses and maxilla studies offers a more holistic and nuanced view of the underlying mechanisms. This comprehensive approach not only aids in discovering new research avenues but also facilitates the identification of potential diagnostic and therapeutic markers (Jurdziński et al., 2020). Focusing exclusively on gingival tissue could potentially overlook the broader, systemic repercussions of the disease. By including the maxilla and blood analyses, researchers are better equipped to develop a thorough understanding of the disease’s impacts, which could lead to more effective diagnostic tools and treatments (Qasim et al., 2020).

The prevalence of a high number of hypomethylated regions in Balb/c mice underlines the fundamental influence of genetic and epigenetic backgrounds in increasing their susceptibility to periodontal disease. Furthermore, variations in the epigenetic control of active and promoter regions between the two lineages potentially contribute to the complex orchestration of immune responses and inflammation. Remarkably, the observed levels of DNA methylation may serve as precursors to the onset of periodontal disease or, conversely, may arise as a result of the disease process. Our study refrains from definitively establishing whether the associations between differential methylation in blood, hemimaxilla and periodontitis are causal or consequential, insisting on the need for further mechanistic research to unravel the temporality and dynamics of these epigenetic changes in the context of periodontal disease.

Our comprehensive pathway analysis has successfully illuminated key biological processes intricately linked to the contrasting susceptibility to periodontal disease between the C57bl/6 (resistant) and Balb/c (susceptible) mouse lineages. Among these processes, the dysregulation of apoptosis, oxidative stress, DNA damage response, TNF signaling via NF-κB, inflammation, PI3K signaling, histone acetylation, and chemokine signaling emerged as pivotal players in shaping the distinct disease susceptibility profiles. Notably, the PI3K/Akt/mTOR signaling pathway, renowned for its multifaceted role in cellular processes encompassing cell growth, proliferation, survival, and metabolism, stands out as a central hub within this intricate network (Hartmann et al., 2021). Its modulation holds particular significance in the context of autophagy, wherein it exerts regulatory control over the initiation and progression of autophagic processes (Sarbassov et al., 2005). The PI3K/AKT pathway is crucial in regulating a myriad of cellular functions, including inflammatory responses. However, its influence extends beyond merely anti-inflammatory activities; it also plays a significant role in activating pro-inflammatory pathways (Manning and Cantley, 2007; Manning and Toker, 2017). The effects of the PI3K/AKT pathway are context-dependent, varying with the type of pathology, the state of the disease, and the cellular conditions. As such, it functions both as a promoter and a suppressor of inflammation, leading to either pathological or protective outcomes in different clinical scenarios (Liu et al., 2009; Holden et al., 2014; Fan et al., 2015). Intriguingly, Franchin M et al.’s (2023) (Franchin et al., 2023) study on periodontal disease variations in Balb/c, C57bl/6, and C57bl/10 mice uncovered compelling correlations. Their findings unveiled reduced bone resorption and diminished pro-inflammatory cytokine levels in C57bl/6-10 mice compared to Balb/c mice, accompanied by heightened activation of the PI3K/Akt pathway and attenuated activation of the mTOR pathway in a ligature-induced periodontitis model (Laplante and Sabatini, 2012; Bi et al., 2023). Corroborating these outcomes, our study employing a gavage model also demonstrated diminished bone resorption in C57bl/6 mice relative to Balb/c mice. These findings underscore the potential anti-inflammatory and cell survival-promoting effects linked to PI3K/Akt pathway activation, as well as the role of mTOR pathway activation in inhibiting autophagy (Lawrence, 2016; Liu et al., 2018). In essence, our pathway analysis unveils the intricate interplay between genetic and stages determinants that collectively underpin susceptibility to periodontal disease.

The PPI network analysis highlighted central proteins, such as Akt1, Ctnnb1 (Beta-catenin), and Hsp90aa1, which are crucial in various cellular functions. Akt1, identified as a highly connected central node, is particularly significant because of its involvement in multiple cellular signaling pathways. It regulates cell growth, survival, and metabolism, indicating its role as a master regulator within the network (Yang et al., 2019; Su et al., 2024). This extensive connectivity underscores the importance of the PI3K/AKT pathway in maintaining cellular homeostasis and responding to external stimuli (Engelman et al., 2006). Ctnnb1, another central node in the network, is essential in the Wnt signaling pathway, where it regulates gene expression and cell adhesion (Manning and Toker, 2017). Its centrality in the network further emphasizes the interconnected nature of these signaling pathways and their collective impact on cellular integrity and function. Hsp90aa1, a heat shock protein critical for protein folding and stability, also plays a vital role in the cellular stress response and protein homeostasis (Manning and Toker, 2017). The presence of such central nodes indicates a highly integrated functional network capable of coordinated responses to external stimuli. Moreover, specific subclusters identified in the PPI network analysis provide insights into particular functional interactions. For instance, the subcluster involving Slc7a1 and Slc3a2 points to a role in amino acid transport, while another subcluster consisting of Cog5, Mtdh, Glod4, and Rab2b suggests functions related to vesicular trafficking and cellular metabolism regulation. Given these findings, the PI3K/AKT signaling pathway’s focus is justified by its central role in regulating vital cellular processes and maintaining cellular homeostasis. This pathway’s critical functions in cell growth, survival, and metabolism make it a key area of interest for further research and potential therapeutic interventions (Yang et al., 2019; Su et al., 2024).

The selection of Reduced Representation Bisulfite Sequencing (RRBS) for DNA methylation analysis is justified by several significant advantages it offers, particularly in the study of specific genomic regions such as promoters and enhancers. RRBS is a targeted sequencing approach that combines restriction enzyme digestion with bisulfite conversion, enabling high-resolution mapping of methylated cytosines at a reduced cost compared to whole-genome bisulfite sequencing (WGBS) (Meissner et al., 2005).A primary reason for choosing RRBS is its enrichment for CpG-rich regions, often located in gene promoters and enhancers. These areas are critical in gene regulation, and their methylation status can significantly impact gene expression (Gu et al., 2011). By focusing on these CpG-dense areas, RRBS provides a detailed and comprehensive methylation profile that is particularly relevant for understanding regulatory mechanisms in the genome.

Additionally, RRBS is highly efficient in terms of sequencing depth and coverage. It allows for the detection of methylation patterns with high precision and sensitivity, even in regions with low levels of methylation (Boyle et al., 2012). This capability is crucial for identifying subtle changes in DNA methylation that may have significant biological implications. Furthermore, RRBS has proven effective in diverse biological samples, providing robust and reproducible results. This method is particularly useful for comparative studies where consistent coverage of CpG sites across samples is essential (Bock et al., 2010).

Our study significantly enhances the understanding of the epigenetic factors involved in periodontal disease, establishing a solid foundation for future investigations into the complex interplay between genetics, epigenetics, and immune responses in the development and progression of this condition. We recognize the limitation of the small sample size (N=3 per group) in this pilot study. However, it is crucial to emphasize that its initial design was explicitly intended to lay a scientific foundation for future research in this area. The choice of a small sample was a strategic decision to guide larger-scale studies, where statistical power and generalization will be prioritized.

## Data availability statement

The raw data supporting the conclusions of this article will be made available by the authors, without undue reservation.

## Ethics statement

The animal study was approved by Ethics Committee on Animal Use of the Faculty of Dentistry of Ribeirão Preto-University of São Paulo The specific protocol number for this study is CEUA/FORP 2018.1.644.58.6. The study was conducted in accordance with the local legislation and institutional requirements.

## Author contributions

CHM: Conceptualization, Data curation, Formal analysis, Funding acquisition, Investigation, Methodology, Project administration, Resources, Software, Supervision, Validation, Visualization, Writing – original draft, Writing – review & editing. JG: Formal analysis, Methodology, Software, Supervision, Validation, Writing – review & editing. LF: Conceptualization, Investigation, Methodology, Visualization, Writing – review & editing. PS: Conceptualization, Investigation, Validation, Visualization, Writing – review & editing. MM: Conceptualization, Methodology, Validation, Visualization, Writing – review & editing. RC: Conceptualization, Data curation, Formal analysis, Methodology, Software, Supervision, Validation, Visualization, Writing – review & editing. HH: Data curation, Formal analysis, Methodology, Software, Supervision, Visualization, Writing – review & editing. DP: Conceptualization, Data curation, Formal analysis, Funding acquisition, Investigation, Methodology, Project administration, Resources, Software, Supervision, Validation, Visualization, Writing – original draft, Writing – review & editing.
