# Supplementary figures and images for: Methylome-wide analysis in systemic microbial-induced experimental periodontal disease in mice with different susceptibility
Source: Front Cell Infect Microbiol. 2024 Jul 16;14:1369226. doi: 10.3389/fcimb.2024.1369226 (PMC11289848; doi:10.3389/fcimb.2024.1369226)

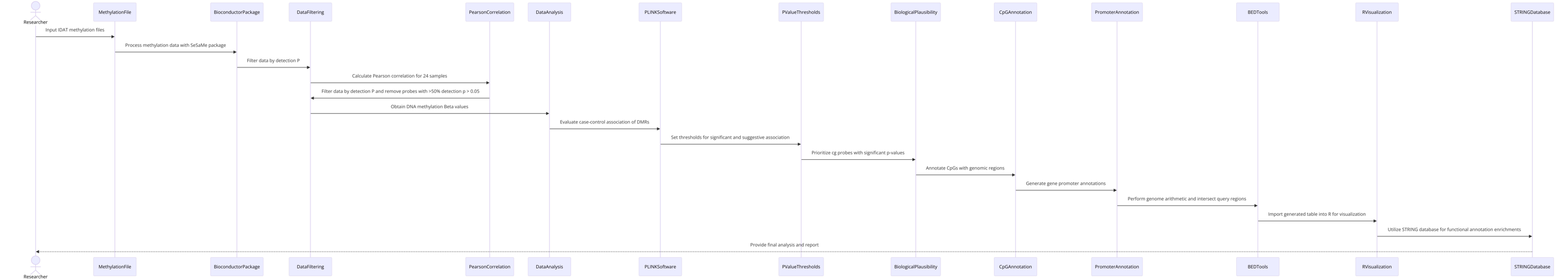

Supplement: Supplementary file 1 [file Image_1.jpeg]
