# Supplementary material for: Methylome-wide analysis in systemic microbial-induced experimental periodontal disease in mice with different susceptibility
Source: Front Cell Infect Microbiol. 2024 Jul 16;14:1369226. doi: 10.3389/fcimb.2024.1369226 (PMC11289848; doi:10.3389/fcimb.2024.1369226)
Supplement: Supplementary file 2 [file Table_1.pdf]

# *Supplementary Material*

## Methylome-wide Analysis in Systemic Microbial-Induced Experimental Periodontal Disease in Mice with Different Susceptibility

**Cristhiam de Jesus Hernandez Martinez**[1,2], **Joseph Glessner**[2,3,4], **Livia Sertori Finoti**[7], **Pedro Felix Silva**[1], **Michel Messora**[1], **Ricardo Della Coletta**[8], **Hakon Hakonarson**[2,3,4,5,6*], **Daniela Bazan Palioto**[a*].

[1]Department of Oral & Maxillofacial Surgery and Periodontology, Ribeirão Preto Dental School, University of São Paulo - USP, Avenida do Café, s/n - FORP-USP, Ribeirão Preto, 14040-904, São Paulo, Brazil.

[2]The Center for Applied Genomics, Children's Hospital of Philadelphia, Philadelphia, PA 19104, USA.

[3]Department of Pediatrics, The Perelman School of Medicine, University of Pennsylvania, Philadelphia, PA 19104, USA.

[4]Division of Human Genetics, Children's Hospital of Philadelphia, Philadelphia, PA 19104, USA.

[5]Division of Pulmonary Medicine, Children's Hospital of Philadelphia, Philadelphia, PA 19104, USA.

[6]Faculty of Medicine, University of Iceland, Reykjavik, Iceland.

[7]Laboratory of Rebecca Ahrens-Nicklas,Children's Hospital of Philadelphia, Philadelphia, PA 19104, USA

[8]Department of Oral Diagnosis and Graduate Program in Oral Biology, Piracicaba Dental School, University of Campinas, Piracicaba, Brazil.

## *Correspondence:

Daniela Bazan Palioto, MD, PhD, Department of Oral & Maxillofacial Surgery and Periodontology, Ribeirão Preto School of Dentistry, University of São Paulo - USP, Avenida do Café, s/n - FORP-USP, Ribeirão Preto, 14040-904, São Paulo, Brazil. Phone number: +55 (16) 3315-3955 | 3315-3971. Email address: dpalioto@forp.usp.br

Hakon Hakonarson, MD, PhD, Center for Applied Genomics, 3615 Civic Center Boulevard, Abramson Building, Philadelphia, PA 19104. Email: hakonarson@chop.edu

**Supplementary Materials and Methods.**

**Supplementary Figure 1.** Bioinformatic Analysis of DNA Methylation Profiles

**Supplementary table 1.** Biological processes and pathways generated by STRING.

**Supplementary table 2.** Most significant biological process.

**Supplementary table 3.** Overrepresented pathway.

**Supplementary Materials and Methods.**

**Representation bisulphite sequencing**

We generated single base resolution DNA methylation data using the Ovation RRBS Methyl-Seq System 1–16 (Tecan Life Sciences) with 100ng of input DNA. This approach employs the MspI restriction enzyme to digest DNA at the CCGG motif, which is highly enriched in CpG dense regions of the genome. This generates a library of small (>300bp) fragments that are rich in CpG content. Sequencing libraries are generated from these fragments, bisulphite converted and sequenced. For this study, we sequenced these libraries 100bp reads per sample on an Illumina NovaSeq instrument (Supplementary Figure 1).

**Mouse methylation BeadChip**

Genome-wide DNA methylation was performed using the Illumina Infinium Mouse Methylation BeadChip (Illumina, San Diego, CA, USA) following the standard manufacturer's protocol. 500ng of high-quality genomic DNA was bisulfite converted using the Zymo EZ-96 DNA Methylation kit (Zymo Research, Irvine, CA, USA). Bisulfite converted samples were then amplified, fragmented, purified and hybridized onto the Mouse Methylation BeadChip. All experiments were conducted following the manufacturer`s protocols by the Centro de Genômica Funcional ESALQ/USP. The arrays were washed and scanned using the Methylation NXT System and then preprocessed by the Illumina GenomeStudio software (Genome Studio Methylation Module v1.8 User Guide) (1).

Array data analysis was performed using R v.4.2.2(2) using packages from Bioconductor (3,4) and other repositories described below (Supplementary Figure 1)

**Methylation array processing**

We used the BiocManager Bioconductor package v.1.30.19 (5) to input the IDAT methylation files outputted by Illumina iScan Software and processed the methylation data using Bioconductor *SeSaMe* package (6) that implement a new method, called *pOOBAH* (*P*-value with out-of-band (OOB) array hybridization) to generate Beta values ($P \geq 0.05$). This method better addresses detection failure (false negatives) as part of a single-sample-based pipeline for DNA methylation, including preprocessing, quality control, visualization, and inference (7). Data was subsequently filtered by detection *P*.

**Differential Methylome**

Pearson correlation was calculated for all 24 samples by selecting the most variably methylated probes. For detection *P* filtering, we masked values that had a detection $p > 0.05$ and removed the probe entirely if >50% of samples had a detection $p > 0.05$. The DNA methylation values, described as DNA methylation Beta values, are continuous variables between 0 and 1, representing the ratio between the intensity of the methylation signal and the intensity of the locus combined. Thus, higher beta values represent a higher level of DNA methylation (hypermethylation), and lower beta values represent a lower-level of DNA methylation, hypomethylation.

The case-control association of the DMRs finding set was evaluated using PLINK version 2.0 software(8). We imported the "dosage" genotypes from the Betas matrix generated by Sesame (continuous values 0 to 1 vs. discrete values AA, AB, BB) into a plink2 format pfile (*.pgen, *.pvar, and *.psam). The strengths for quantitative phenotypes were assessed using '**firth**' logistic regression analysis (9) and reported as odds ratio (OR) which calculated with their 95% confidence intervals (CIs) after filtering the methylation levels of each cg probes based on the control population, quantitative trait loci(QTL) 0.084 and *P* value; *P* values of less than $1.00 \times 10^{-8}$ and $5.00 \times 10^{-5}$ ($p<0.05$) were required for significant association and suggestive association, respectively. However, the power for statistical association is limited strongly by the small sample size.

Biological plausibility in relation to their phenotype of interest was considered to prioritize cg probes of significant p-value based on Illumina Methylation array annotation Infinium Mouse Methylation BeadChip Product Files (illumina.com) we specifically used Gene Probe Annotation File (CSV) for the Infinium Mouse Methylation array and Infinium Mouse Methylation Manifest File.

**Genomic region annotations of the DMRs (p<0.05 cg probes)**

We note the DMRs for each sample group comparison result (C57bl/6C vs C57bl/6-*Pg*-induced; Balb/cC vs Balb/c-*Pg*-induced; C57bl/6-*Pg*-induced vs Balb/c-*Pg*-induced) and compare them to the whole genome definition files from chromosome analysis and cg probe positions. To annotate CpGs with respect to whether they reside in CpG islands, we downloaded the mm10 cpgIslandExt table from the UCSC table browser. The cpgIslandExt table contains annotations of genomic region of CpG islands, meeting the following criteria: having >50% GC content, a length of >200bp and a ratio of observed to expected CG dinucleotides > 0.6.

Annotations of candidate regulatory elements were downloaded from the murine ENCODE project web portal (10). We examined overlaps between CpGs and candidate regulatory elements using the intersect function of BEDTools (v2.29.0).

Repetitive elements were annotated. Briefly, pre-computed annotations for the mm10 genome were downloaded and BEDTools was used to find the overlap between these repetitive elements and CpGs in datasets.

Gene promoter annotations were generated using the mm10 reference genome. CpGs that were subsequently annotated as promoter and ascribed to a gene were used for downstream gene promoter analysis.

For this purpose, a powerful toolset for genome arithmetic BEDTools software was used in *Linux* compute environment (11).

Mouse Genome Annotation Beds Analyzed:

- **TSS transcription start sites**

https://refoss.riken.jp/datafiles/current/mouse/

refTSS_v3.3_mouse_coordinate.mm10.bed.gz

- **Promoter regions**

UCSC Table Browser mm10

https://genome.ucsc.edu/cgi-bin/hgTables?clade=mammal&org=Mouse&db=mm10

group: Expression and Regulation

track: EPDnew Promoters

table: EPDnew v6 (epdNewPromoter)

- **Enhancer (potentiator) regions**

UCSC Table Browser mm10

https://genome.ucsc.edu/cgi-bin/hgTables?clade=mammal&org=Mouse&db=mm10

group: Expression and Regulation

track: ENC+EPD Enhc-Gene

table: Enhancers Rep (egEnhancerRep)

- **<u>CpG site types</u>**

group: Expression and Regulation

track: CpG Islands

table: cpgIslandExt

BEDTools intersections were made for each of the mouse genome annotation beds for the query regions (significant association cg probes), thus generating a table that was then imported into R to generate graphs to visualize the data.

**Functional annotation enrichments**

The STRING database (https://string-db.org/) was utilized for analyzing functional annotation enrichments of multiple proteins implicated by our study(12) (Supplementary Figure 1)

## References

1. Teschendorff AE, Relton CL. Statistical and integrative system-level analysis of DNA methylation data. Vol. 19, Nature Reviews Genetics. 2018.
2. R Core Team. R Core Team 2021 R: A language and environment for statistical computing. R foundation for statistical computing. https://www.R-project.org/. R Found Stat Comput. 2022;2.
3. Huber W, Carey VJ, Gentleman R, Anders S, Carlson M, Carvalho BS, et al. Orchestrating high-throughput genomic analysis with Bioconductor. Nat Methods. 2015;12(2).
4. Kirill Müller. here: A Simpler Way to Find Your Files. 2020.
5. Morgan. BiocVersion: Set the appropriate version of Bioconductor packages. 2023.
6. Zhou W, Triche TJ, Laird PW, Shen H. SeSAMe: Reducing artifactual detection of DNA methylation by Infinium BeadChips in genomic deletions. Nucleic Acids Res. 2018;46(20).
7. Triche TJ, Weisenberger DJ, Van Den Berg D, Laird PW, Siegmund KD. Low-level processing of Illumina Infinium DNA Methylation BeadArrays. Nucleic Acids Res. 2013;41(7).
8. Chang CC, Chow CC, Tellier LCAM, Vattikuti S, Purcell SM, Lee JJ. Second-generation PLINK: Rising to the challenge of larger and richer datasets. Gigascience. 2015;4(1).
9. Ma C, Blackwell T, Boehnke M, Scott LJ. Recommended joint and meta-analysis strategies for case-control association testing of single low-count variants. Genet Epidemiol. 2013;37(6).
10. Stamatoyannopoulos JA, Snyder M, Hardison R, Ren B, Gingeras T, Gilbert DM, et al. An encyclopedia of mouse DNA elements (Mouse ENCODE). Vol. 13, Genome Biology. 2012.
11. Quinlan AR, Hall IM. BEDTools: A flexible suite of utilities for comparing genomic features. Bioinformatics. 2010;26(6).
12. Szklarczyk D, Kirsch R, Koutrouli M, Nastou K, Mehryary F, Hachilif R, et al. The STRING database in 2023: protein-protein association networks and functional enrichment analyses for any sequenced genome of interest. Nucleic Acids Res. 2023;51(1 D).

**Supplementary table 1.** Biological processes and pathways generated by STRING

| Term ID | Term description | Observed gene count | Background gene count | False discovery rate | Matching proteins in your network (labels) |
|---|---|---|---|---|---|
| GO:0046907 | Intracellular transport | 14 | 1166 | 0.0058 | Akt1,Map2k1,Napa,Bcl2l1,Nutf2,Syngr1,Rab2b,Ywhaz,Arl8a,Eif5a,Hsp90aa1,Atp5d,Myo1c,Aktip |
| GO:0051052 | Regulation of DNA metabolic process | 9 | 365 | 0.0058 | Akt1,Msh6,Ctnnb1,Prkdc,Msh2,Sh2b1,Hsp90aa1,Klf4,Rad51ap1 |
| GO:0051641 | Cellular localization | 19 | 2115 | 0.0058 | Akt1,Map2k1,Napa,Ctnnb1,Bcl2l1,Nutf2,Syngr1,Rab2b,Ywhaz,Msh2,Arl8a,Brat1,Eif5a,Lamtor4,Hsp90aa1,Atp5d,Myo1c,Wdr45,Aktip |
| GO:0051649 | Establishment of localization in cell | 15 | 1518 | 0.0171 | Akt1,Map2k1,Napa,Ctnnb1,Bcl2l1,Nutf2,Syngr1,Rab2b,Ywhaz,Arl8a,Eif5a,Hsp90aa1,Atp5d,Myo1c,Aktip |
| GO:0071704 | Organic substance metabolic process | 34 | 6824 | 0.0190 | Akt1,Map2k1,Msh6,Psmb4,Ctnnb1,Ppp2r1a,Ppp2ca,Pcbd2,Ywhaz,Prkdc,Msh2,Cyb5r1,Trim33,Brat1,Cyp2s1,Eif5a,Suclg1,Cpsf6,Afmid,Hsp90aa1,Pigt,Atp5d,Klf4,Ldha,Pnpla6,Rad51ap1,Wdr45,Hecw2,Aktip,Psma3,Tecr,Slc3a2,Fam96b,Aldh7a1 |
| GO:0007346 | Regulation of mitotic cell cycle | 9 | 531 | 0.0197 | Akt1,Map2k1,Ctnnb1,Bcl2l1,Prkdc,Msh2,Sh2b1,Klf4,Hecw2 |
| GO:0008152 | Metabolic process | 35 | 7331 | 0.0236 | Akt1,Map2k1,Msh6,Psmb4,Ctnnb1,Ppp2r1a,Ppp2ca,Pcbd2,Ywhaz,Prkdc,Pdia5,Msh2,Cyb5r1,Trim33,Brat1,Cyp2s1,Eif5a,Suclg1,Cpsf6,Afmid,Hsp90aa1,Pigt,Atp5d,Klf4,Ldha,Pnpla6,Rad51ap1,Wdr45,Hecw2,Aktip,Psma3,Tecr,Slc3a2,Fam96b,Aldh7a1 |
| GO:0008104 | Protein localization | 16 | 1890 | 0.0258 | Akt1,Napa,Ctnnb1,Nutf2,Syngr1,Rab2b,Ywhaz,Msh2,Arl8a,Cog5,Eif5a,Lamtor4,Hsp90aa1,Myo1c,Wdr45,Aktip |
| GO:0044238 | Primary metabolic process | 32 | 6369 | 0.0258 | Akt1,Map2k1,Msh6,Psmb4,Ctnnb1,Ppp2r1a,Ppp2ca,Ywhaz,Prkdc,Msh2,Cyb5r1,Trim33,Brat1,Cyp2s1,Eif5a,Suclg1,Cpsf6,Afmid,Hsp90aa1,Pigt,Atp5d,Klf4,Ldha,Pnpla6,Rad51ap1,Wdr45,Hecw2,Aktip,Psma3,Tecr,Slc3a2,Fam96b |
| GO:0051054 | Positive regulation of DNA metabolic process | 6 | 212 | 0.0264 | Akt1,Ctnnb1,Prkdc,Msh2,Hsp90aa1,Klf4 |
| GO:0009987 | Cellular process | 49 | 13330 | 0.0302 | S100a6,Akt1,Map2k1,Msh6,Psmb4,Napa,Ctnnb1,Ppp2r1a,Bcl2l1,Nutf2,Syngr1,Ppp2ca,Pcbd2,Rab2b,Mtdh,Ywhaz,Prkdc,Pdia5,Msh2,Arl8a,Trim33,Sh2b1,Brat1,Cyp2s1,Cog5,Fmnl1,Slc7a1,Eif5a,Lamtor4,Suclg1,Cpsf6,Afmid,Hsp90aa1,Scrt1,Pigt,Atp5d,Klf4,Ldha,Myo1c,Pnpla6,Rad51ap1, Wdr45,Hecw2,Aktip,Psma3,Tecr,Slc3a2,Fam96b,Aldh7a1 |
| GO:0071705 | Nitrogen compound transport | 14 | 1534 | 0.0305 | Akt1,Napa,Nutf2,Syngr1,Rab2b,Ywhaz,Arl8a,Cog5,Slc7a1,Eif5a,Hsp90aa1,Myo1c,Aktip,Slc3a2 |
| GO:0006807 | Nitrogen compound metabolic process | 29 | 5878 | 0.0457 | Akt1,Map2k1,Msh6,Psmb4,Ctnnb1,Ppp2r1a,Ppp2ca,Pcbd2,Ywhaz,Prkdc,Msh2,Trim33,Eif5a,Suclg1,Cpsf6,Afmid,Hsp90aa1,Pigt,Atp5d,Klf4,Pnpla6,Rad51ap1,Wdr45,Hecw2,Aktip,Psma3,Tecr,Fam96b,Aldh7a1 |
| GO:0006915 | Apoptotic process | 10 | 825 | 0.0457 | Akt1,Msh6,Bcl2l1,Prkdc,Msh2,Brat1,Eif5a,Hsp90aa1,Pigt,Aktip |
| GO:0008630 | Intrinsic apoptotic signaling pathway in response to DNA damage | 4 | 75 | 0.0457 | Msh6,Bcl2l1,Prkdc,Msh2 |
| GO:0010212 | Response to ionizing radiation | 5 | 151 | 0.0457 | Bcl2l1,Prkdc,Msh2,Brat1,Rad51ap1 |
| GO:0015031 | Protein transport | 12 | 1204 | 0.0457 | Akt1,Napa,Nutf2,Syngr1,Rab2b,Ywhaz,Arl8a,Cog5,Eif5a,Hsp90aa1,Myo1c,Aktip |
| GO:0016447 | Somatic recombination of immunoglo | 3 | 26 | 0.0457 | Msh6,Prkdc,Msh2 |

| | | | | | |
|---|---|---|---|---|---|
| | bulin gene segments | | | | |
| GO:0034613 | Cellular protein localization | 13 | 1392 | 0.0457 | Akt1,Napa,Ctnnb1,Nutf2,Syngr1,Rab2b,Ywhaz,Msh2,Eif5a,Lamtor4,Hsp90aa1,Myo1c,Wdr45 |
| GO:0044237 | Cellular metabolic process | 31 | 6445 | 0.0457 | Akt1,Map2k1,Msh6,Psmb4,Ctnnb1,Ppp2r1a,Ppp2ca,Pcbd2,Ywhaz,Prkdc,Msh2,Trim33,Cyp2s1,Eif5a,Suclg1,Cpsf6,Afmid,Hsp90aa1,Pigt,Atp5d,Klf4,Ldha,Pnpla6,Rad51ap1,Wdr45,Hecw2,Aktip,Psma3,Tecr,Fam96b,Aldh7a1 |
| GO:0046898 | Response to cycloheximide | 2 | 3 | 0.0457 | Bcl2l1,Klf4 |
| GO:0051726 | Regulation of cell cycle | 11 | 1016 | 0.0457 | Akt1,Map2k1,Ctnnb1,Ppp2r1a,Bcl2l1,Ppp2ca,Prkdc,Msh2,Sh2b1,Klf4,Hecw2 |
| GO:0051973 | Positive regulation of telomerase activity | 3 | 34 | 0.0479 | Ctnnb1,Hsp90aa1,Klf4 |
| GO:0055114 | Oxidation-reduction process | 10 | 917 | 0.0479 | Akt1,Pcbd2,Pdia5,Cyb5r1,Cyp2s1,Suclg1,Atp5d,Ldha,Tecr,Aldh7a1 |
| GO:1903827 | Regulation of cellular protein localization | 8 | 576 | 0.0479 | Akt1,Map2k1,Ctnnb1,Bcl2l1,Nutf2,Ppp2ca,Hsp90aa1,Myo1c |
| GO:0006886 | Intracellular protein transport | 9 | 759 | 0.0488 | Akt1,Napa,Nutf2,Syngr1,Rab2b,Ywhaz,Eif5a,Hsp90aa1,Myo1c |
| GO:0010564 | Regulation of cell cycle process | 8 | 583 | 0.0488 | Akt1,Ctnnb1,Ppp2r1a,Bcl2l1,Prkdc,Sh2b1,Klf4,Hecw2 |
| GO:1900748 | Positive regulation of vascular endothelial growth factor signaling pathway | 2 | 5 | 0.0488 | Myo1c,Ccbe1 |
| GO:0019899 | Enzyme binding | 19 | 2277 | 0.0070 | Akt1,Map2k1,Msh6,Ctnnb1,Bcl2l1,Nutf2,Ppp2ca,Ywhaz,Prkdc,Msh2,Sh2b1,Fmnl1,Lamtor4,Hsp90aa1,Klf4,Myo1c,Wdr45,Ccbe1,Psma3 |
| GO:0044877 | Protein-containing complex binding | 15 | 1411 | 0.0070 | Map2k1,Msh6,Napa,Ctnnb1,Bcl2l1,Ppp2ca,Ywhaz,Msh2,Fmnl1,Eif5a,Suclg1,Cpsf6,Atp5d,Myo1c,Ccbe1 |
| GO:0019901 | Protein kinase binding | 10 | 707 | 0.0142 | Akt1,Map2k1,Ctnnb1,Bcl2l1,Ppp2ca,Ywhaz,Msh2,Sh2b1,Hsp90aa1,Wdr45 |
| GO:0032142 | Single guanine insertion binding | 2 | 2 | 0.0278 | Msh6,Msh2 |
| GO:0032143 | Single thymine insertion binding | 2 | 2 | 0.0278 | Msh6,Msh2 |
| GO:0003824 | Catalytic activity | 27 | 5224 | 0.0300 | Akt1,Map2k1,Msh6,Psmb4,Ppp2r1a,Ppp2ca,Pcbd2,Rab2b,Prkdc,Pdia5,Msh2,Cyb5r1,Trim33,Cyp2s1,Suclg1,Afmid,Hsp90aa1,Atp5d,Ldha,Myo1c,Pnpla6,Hecw2,Aktip,Psma3,Tecr,Slc3a2,Aldh7a1 |
| GO:0032137 | Guanine/thymine mispair binding | 2 | 3 | 0.0300 | Msh6,Msh2 |
| GO:0043227 | Membrane-bounded organelle | 48 | 10370 | 1.17e-05 | S100a6,Akt1,Map2k1,Msh6,Psmb4,Napa,Ctnnb1,Ppp2r1a,Bcl2l1,Nutf2,Syngr1,Glod4,Ppp2ca,Pcbd2,Rab2b,Mtdh,Ywhaz,Prkdc,Pdia5,Msh2,Arl8a,Cyb5r1,Trim33,Sh2b1,Brat1,Cyp2s1,Cog5,Fmnl1,Eif5a,Lamtor4,Suclg1,Cpsf6,Afmid,Hsp90aa1,Scrt1,Pigt,Atp5d,Klf4,Ldha,Myo1c,Pnpla6, Rad51ap1,Aktip,Psma3,Tecr,Slc3a2,Fam96b,Aldh7a1 |

| | | | | | |
|---|---|---|---|---|---|
| GO:0043229 | Intracellular organelle | 49 | 11084 | 1.17e-05 | S100a6,Akt1,Map2k1,Msh6,Psmb4,Napa,Ctnnb1,Ppp2r1a,Bcl2l1,Nutf2,Syngr1,Glod4,Ppp2ca,Pcbd2,Rab2b,Mtdh,Ywhaz,Prkdc,Pdia5,Msh2,Arl8a,Cyb5r1,Trim33,Sh2b1,Brat1,Cyp2s1,Cog5,Fmnl1,Eif5a,Lamtor4,Suclg1,Cpsf6,Afmid,Hsp90aa1,Scrt1,Pigt,Atp5d,Klf4,Ldha,Myo1c,Pnpla6, Rad51ap1,Hecw2,Aktip,Psma3,Tecr,Slc3a2,Fam96b,Aldh7a1 |
| GO:0043231 | Intracellular membrane-bounded organelle | 46 | 9507 | 1.17e-05 | S100a6,Akt1,Map2k1,Msh6,Psmb4,Napa,Ctnnb1,Ppp2r1a,Bcl2l1,Nutf2,Glod4,Ppp2ca,Pcbd2,Rab2b,Mtdh,Ywhaz,Prkdc,Pdia5,Msh2,Arl8a,Cyb5r1,Trim33,Sh2b1,Brat1,Cyp2s1,Cog5,Eif5a,Lamtor4,Suclg1,Cpsf6,Afmid,Hsp90aa1,Scrt1,Pigt,Atp5d,Klf4,Ldha,Myo1c,Pnpla6,Rad51ap1,Aktip, Psma3,Tecr,Slc3a2,Fam96b,Aldh7a1 |
| GO:0005622 | Intracellular | 50 | 12596 | 0.00018 | S100a6,Akt1,Map2k1,Msh6,Psmb4,Napa,Ctnnb1,Ppp2r1a,Bcl2l1,Nutf2,Syngr1,Glod4,Ppp2ca,Pcbd2,Rab2b,Mtdh,Ywhaz,Prkdc,Pdia5,Msh2,Arl8a,Cyb5r1,Trim33,Sh2b1,Brat1,Cyp2s1,Cog5,Fmnl1,Eif5a,Lamtor4,Suclg1,Cpsf6,Afmid,Hsp90aa1,Scrt1,Pigt,Atp5d,Klf4,Ldha,Myo1c,Pnpla6, Rad51ap1,Wdr45,Hecw2,Aktip,Psma3,Tecr,Slc3a2,Fam96b,Aldh7a1 |
| GO:0005737 | Cytoplasm | 45 | 10283 | 0.00018 | S100a6,Akt1,Map2k1,Msh6,Psmb4,Napa,Ctnnb1,Ppp2r1a,Bcl2l1,Nutf2,Syngr1,Glod4,Ppp2ca,Pcbd2,Rab2b,Mtdh,Ywhaz,Pdia5,Arl8a,Cyb5r1,Sh2b1,Brat1,Cyp2s1,Cog5,Fmnl1,Eif5a,Lamtor4,Suclg1,Cpsf6,Afmid,Hsp90aa1,Pigt,Atp5d,Klf4,Ldha,Myo1c,Pnpla6,Wdr45,Hecw2,Aktip,Psma3,Tecr,Slc3a2,Fam96b,Aldh7a1 |
| GO:0005829 | Cytosol | 25 | 3573 | 0.00018 | S100a6,Akt1,Map2k1,Msh6,Psmb4,Ctnnb1,Ppp2r1a,Bcl2l1,Nutf2,Ppp2ca,Ywhaz,Sh2b1,Cog5,Fmnl1,Suclg1,Afmid,Hsp90aa1,Ldha,Myo1c,Wdr45,Aktip,Psma3,Slc3a2,Fam96b,Aldh7a1 |
| GO:0032991 | Protein-containing complex | 30 | 4895 | 0.00018 | Akt1,Msh6,Psmb4,Napa,Ctnnb1,Ppp2r1a,Bcl2l1,Nutf2,Ppp2ca,Ywhaz,Prkdc,Msh2,Cog5,Slc7a1,Eif5a,Lamtor4,Suclg1,Cpsf6,Hsp90aa1,Pigt,Atp5d,Klf4,Myo1c,Creg1,Rad51ap1,Aktip,Ccbe1,Psma3,Slc3a2,Fam96b |
| GO:0005634 | Nucleus | 32 | 6330 | 0.0026 | S100a6,Akt1,Map2k1,Msh6,Psmb4,Ctnnb1,Ppp2r1a,Bcl2l1,Nutf2,Ppp2ca,Pcbd2,Mtdh,Ywhaz,Prkdc,Msh2,Trim33,Sh2b1,Brat1,Cog5,Eif5a,Cpsf6,Afmid,Hsp90aa1,Scrt1,Klf4,Myo1c,Rad51ap1,Aktip,Psma3,Slc3a2,Fam96b,Aldh7a1 |
| GO:0032301 | MutSalpha complex | 2 | 2 | 0.0066 | Msh6,Msh2 |
| GO:0110165 | Cellular anatomical entity | 53 | 15632 | 0.0066 | S100a6,Akt1,Map2k1,Msh6,Psmb4,Napa,Ctnnb1,Ppp2r1a,Bcl2l1,Nutf2,Syngr1,Glod4,Ppp2ca,Pcbd2,Rab2b,Mtdh,Ywhaz,Prkdc,Pdia5,Msh2,Arl8a,Cyb5r1,Trim33,Sh2b1,Brat1,Cyp2s1,Cog5,Fmnl1,Slc7a1,Eif5a,Lamtor4,Suclg1,Cpsf6,Afmid,Hsp90aa1,Scrt1,Pigt,Atp5d,Klf4,Ldha,Myo1c,Pnpla6, Creg1,Rad51ap1,Wdr45,Hecw2,Aktip,Ccbe1,Psma3,Tecr,Slc3a2,Fam96b,Aldh7a1 |
| GO:0045202 | Synapse | 13 | 1492 | 0.0132 | Map2k1,Napa,Ctnnb1,Ppp2r1a,Bcl2l1,Syngr1,Ppp2ca,Rab2b,Ywhaz,Arl8a,Eif5a,Psma3,Slc3a2 |
| GO:0031090 | Organelle membrane | 19 | 2973 | 0.0142 | Napa,Ctnnb1,Bcl2l1,Nutf2,Syngr1,Rab2b,Mtdh,Arl8a,Cyp2s1,Cog5,Eif5a,Lamtor4,Suclg1,Pigt,Atp5d,Myo1c,Pnpla6,Tecr,Slc3a2 |
| GO:1990391 | DNA repair complex | 3 | 33 | 0.0142 | Msh6,Prkdc,Msh2 |
| GO:0005635 | Nuclear envelope | 7 | 455 | 0.0172 | S100a6,Ctnnb1,Bcl2l1,Nutf2,Mtdh,Eif5a,Myo1c |
| GO:0030054 | Cell junction | 15 | 2050 | 0.0172 | Akt1,Map2k1,Napa,Ctnnb1,Ppp2r1a,Bcl2l1,Syngr1,Ppp2ca,Rab2b,Mtdh,Ywhaz,Arl8a,Eif5a,Psma3,Slc3a2 |
| GO:0042470 | Melanosome | 4 | 102 | 0.0172 | Syngr1,Ywhaz,Hsp90aa1,Slc3a2 |
| GO:0098805 | Whole membrane | 12 | 1415 | 0.0198 | Napa,Ctnnb1,Bcl2l1,Nutf2,Syngr1,Ppp2ca,Arl8a,Eif5a,Lamtor4,Myo1c,Wdr45,Slc3a2 |
| GO:0005819 | Spindle | 6 | 352 | 0.0253 | Akt1,Ctnnb1,Ppp2ca,Arl8a,Hecw2,Fam96b |
| GO:0005643 | Nuclear pore | 3 | 62 | 0.0488 | Nutf2,Eif5a,Myo1c |
| mmu05160 | Hepatitis C | 6 | 158 | 0.0013 | Akt1,Map2k1,Ctnnb1,Ppp2r1a,Ppp2ca,Ywhaz |
| mmu05210 | Colorectal cancer | 5 | 88 | 0.0013 | Akt1,Map2k1,Msh6,Ctnnb1,Msh2 |
| mmu04730 | Long-term depression | 4 | 58 | 0.0022 | Map2k1,Ppp2r1a,Ppp2ca,Gucy1a2 |
| mmu04151 | PI3K-Akt signaling pathway | 7 | 355 | 0.0032 | Akt1,Map2k1,Ppp2r1a,Bcl2l1,Ppp2ca,Ywhaz,Hsp90aa1 |
| mmu01524 | Platinum drug resistance | 4 | 77 | 0.0038 | Akt1,Msh6,Bcl2l1,Msh2 |
| mmu05215 | Prostate cancer | 4 | 99 | 0.0080 | Akt1,Map2k1,Ctnnb1,Hsp90aa1 |
| mmu04114 | Oocyte meiosis | 4 | 115 | 0.0120 | Map2k1,Ppp2r1a,Ppp2ca,Ywhaz |
| mmu04071 | Sphingolipid signaling pathway | 4 | 122 | 0.0131 | Akt1,Map2k1,Ppp2r1a,Ppp2ca |

| | | | | | |
|---|---|---|---|---|---|
| mmu05010 | Alzheimer disease | 6 | 359 | 0.0131 | Akt1,Map2k1,Psmb4,Ctnnb1,Atp5d,Psma3 |
| mmu05200 | Pathways in cancer | 7 | 528 | 0.0144 | Akt1,Map2k1,Msh6,Ctnnb1,Bcl2l1,Msh2,Hsp90aa1 |
| mmu04140 | Autophagy - animal | 4 | 137 | 0.0145 | Akt1,Map2k1,Bcl2l1,Ppp2ca |
| mmu04550 | Signaling pathways regulating pluripotency of stem cells | 4 | 137 | 0.0145 | Akt1,Map2k1,Ctnnb1,Klf4 |
| mmu05213 | Endometrial cancer | 3 | 58 | 0.0145 | Akt1,Map2k1,Ctnnb1 |
| mmu04150 | mTOR signaling pathway | 4 | 156 | 0.0184 | Akt1,Map2k1,Lamtor4,Slc3a2 |
| mmu04390 | Hippo signaling pathway | 4 | 156 | 0.0184 | Ctnnb1,Ppp2r1a,Ppp2ca,Ywhaz |
| mmu05230 | Central carbon metabolism in cancer | 3 | 69 | 0.0184 | Akt1,Map2k1,Ldha |
| mmu05212 | Pancreatic cancer | 3 | 74 | 0.0198 | Akt1,Map2k1,Bcl2l1 |
| mmu05220 | Chronic myeloid leukemia | 3 | 74 | 0.0198 | Akt1,Map2k1,Bcl2l1 |
| mmu05225 | Hepatocellular carcinoma | 4 | 168 | 0.0198 | Akt1,Map2k1,Ctnnb1,Bcl2l1 |
| mmu01521 | EGFR tyrosine kinase inhibitor resistance | 3 | 78 | 0.0200 | Akt1,Map2k1,Bcl2l1 |
| mmu03430 | Mismatch repair | 2 | 22 | 0.0275 | Msh6,Msh2 |
| mmu04914 | Progesterone-mediated oocyte maturation | 3 | 90 | 0.0275 | Akt1,Map2k1,Hsp90aa1 |
| mmu05165 | Human papillomavirus infection | 5 | 342 | 0.0287 | Akt1,Map2k1,Ctnnb1,Ppp2r1a,Ppp2ca |
| mmu03015 | mRNA surveillance pathway | 3 | 96 | 0.0296 | Ppp2r1a,Ppp2ca,Cpsf6 |
| mmu05132 | Salmonella infection | 4 | 213 | 0.0315 | Akt1,Map2k1,Ctnnb1,Hsp90aa1 |
| mmu05142 | Chagas disease | 3 | 100 | 0.0315 | Akt1,Ppp2r1a,Ppp2ca |
| mmu04066 | HIF-1 signaling pathway | 3 | 111 | 0.0393 | Akt1,Map2k1,Ldha |
| mmu04722 | Neurotrophin signaling pathway | 3 | 116 | 0.0423 | Akt1,Map2k1,Sh2b1 |
| mmu05012 | Parkinson disease | 4 | 239 | 0.0423 | Psmb4,Bcl2l1,Atp5d,Psma3 |
| mmu00640 | Propanoate metabolism | 2 | 34 | 0.0427 | Suclg1,Ldha |
| mmu04919 | Thyroid hormone signaling pathway | 3 | 119 | 0.0427 | Akt1,Map2k1,Ctnnb1 |

| | | | | | |
|---|---|---|---|---|---|
| mmu04152 | AMPK signaling pathway | 3 | 123 | 0.0439 | Akt1,Ppp2r1a,Ppp2ca |
| mmu05216 | Thyroid cancer | 2 | 36 | 0.0439 | Map2k1,Ctnnb1 |
| mmu04728 | Dopaminergic synapse | 3 | 127 | 0.0450 | Akt1,Ppp2r1a,Ppp2ca |
| mmu00620 | Pyruvate metabolism | 2 | 39 | 0.0466 | Ldha,Aldh7a1 |
| mmu04915 | Estrogen signaling pathway | 3 | 130 | 0.0466 | Akt1,Map2k1,Hsp90aa1 |
| mmu04210 | Apoptosis | 3 | 135 | 0.0489 | Akt1,Map2k1,Bcl2l1 |
| MMU-201681 | TCF dependent signaling in response to WNT | 7 | 148 | 0.00023 | Akt1,Psmb4,Ctnnb1,Ppp2r1a,Ppp2ca,Ywhaz,Psma3 |
| MMU-195253 | Degradation of beta-catenin by the destruction complex | 5 | 77 | 0.0018 | Psmb4,Ctnnb1,Ppp2r1a,Ppp2ca,Psma3 |
| MMU-168256 | Immune System | 15 | 1621 | 0.0040 | Akt1,Psmb4,Ctnnb1,Ppp2r1a,Bcl2l1,Syngr1,Ppp2ca,Ywhaz,Arl8a,Sh2b1,Hsp90aa1,Myo1c,Creg1,Hecw2,Psma3 |
| MMU-196299 | Beta-catenin phosphorylation cascade | 3 | 15 | 0.0040 | Ctnnb1,Ppp2r1a,Ppp2ca |
| MMU-69206 | G1/S Transition | 5 | 106 | 0.0040 | Akt1,Psmb4,Ppp2r1a,Ppp2ca,Psma3 |
| MMU-1257604 | PIP3 activates AKT signaling | 6 | 234 | 0.0061 | Akt1,Psmb4,Ppp2r1a,Ppp2ca,Lamtor4,Psma3 |
| MMU-168249 | Innate Immune System | 11 | 949 | 0.0061 | Psmb4,Ctnnb1,Ppp2r1a,Bcl2l1,Syngr1,Ppp2ca,Arl8a,Hsp90aa1,Myo1c,Creg1,Psma3 |
| MMU-389513 | CTLA4 inhibitory signaling | 3 | 20 | 0.0061 | Akt1,Ppp2r1a,Ppp2ca |
| MMU-1280215 | Cytokine Signaling in Immune system | 7 | 381 | 0.0089 | Akt1,Psmb4,Ppp2r1a,Ppp2ca,Ywhaz,Sh2b1,Psma3 |
| MMU-450531 | Regulation of mRNA stability by proteins that bind AU-rich elements | 4 | 84 | 0.0089 | Akt1,Psmb4,Ywhaz,Psma3 |
| MMU-4641262 | Disassembly of the destruction complex and recruitment of AXIN to the membrane | 3 | 28 | 0.0089 | Ctnnb1,Ppp2r1a,Ppp2ca |

| | | | | | |
|---|---|---|---|---|---|
| MMU-5218920 | VEGFR2 mediated vascular permeability | 3 | 27 | 0.0089 | Akt1,Ctnnb1,Hsp90aa1 |
| MMU-5673001 | RAF/MAP kinase cascade | 6 | 266 | 0.0089 | Map2k1,Psmb4,Ppp2r1a,Bcl2l1,Ppp2ca,Psma3 |
| MMU-9013694 | Signaling by NOTCH4 | 2 | 3 | 0.0089 | Akt1,Ywhaz |
| MMU-3769402 | Deactivation of the beta-catenin transactivating complex | 3 | 33 | 0.0093 | Akt1,Ctnnb1,Ywhaz |
| MMU-5673000 | RAF activation | 3 | 33 | 0.0093 | Map2k1,Ppp2r1a,Ppp2ca |
| MMU-6804757 | Regulation of TP53 Degradation | 3 | 34 | 0.0093 | Akt1,Ppp2r1a,Ppp2ca |
| MMU-69275 | G2/M Transition | 5 | 180 | 0.0093 | Psmb4,Ppp2r1a,Ppp2ca,Hsp90aa1,Psma3 |
| MMU-5675221 | Negative regulation of MAPK pathway | 3 | 42 | 0.0133 | Map2k1,Ppp2r1a,Ppp2ca |
| MMU-6807070 | PTEN Regulation | 4 | 112 | 0.0138 | Akt1,Psmb4,Lamtor4,Psma3 |
| MMU-5357801 | Programmed Cell Death | 4 | 116 | 0.0151 | Ctnnb1,Bcl2l1,Ywhaz,Hsp90aa1 |
| MMU-212436 | Generic Transcription Pathway | 9 | 834 | 0.0159 | Akt1,Psmb4,Ctnnb1,Ppp2r1a,Ppp2ca,Ywhaz,Trim33,Lamtor4,Psma3 |
| MMU-1474151 | Tetrahydrobiopterin (BH4) synthesis, recycling, salvage and regulation | 2 | 9 | 0.0191 | Akt1,Hsp90aa1 |
| MMU-74160 | Gene expression (Transcription) | 10 | 1055 | 0.0191 | Akt1,Psmb4,Ctnnb1,Ppp2r1a,Ppp2ca,Ywhaz,Trim33,Lamtor4,Myo1c,Psma3 |
| MMU-109582 | Hemostasis | 7 | 524 | 0.0210 | Akt1,Ppp2r1a,Ppp2ca,Ywhaz,Cyb5r1,Sh2b1,Slc3a2 |
| MMU-113501 | Inhibition of replication initiation of damaged DNA by RB1/E2F1 | 2 | 10 | 0.0210 | Ppp2r1a,Ppp2ca |
| MMU-194315 | Signaling by Rho GTPases | 6 | 381 | 0.0217 | Ctnnb1,Ppp2r1a,Ppp2ca,Ywhaz,Fmnl1,Hsp90aa1 |
| MMU-195258 | RHO GTPase Effectors | 5 | 246 | 0.0217 | Ctnnb1,Ppp2r1a,Ppp2ca,Ywhaz,Fmnl1 |
| MMU-203615 | eNOS activation | 2 | 11 | 0.0219 | Akt1,Hsp90aa1 |
| MMU-202670 | ERKs are inactivated | 2 | 12 | 0.0237 | Ppp2r1a,Ppp2ca |

| | | | | | |
|---|---|---|---|---|---|
| MMU-449147 | Signaling by Interleukins | 5 | 257 | 0.0237 | Psmb4,Ppp2r1a,Ppp2ca,Ywhaz,Psma3 |
| MMU-9614399 | Regulation of localization of FOXO transcription factors | 2 | 12 | 0.0237 | Akt1,Ywhaz |
| MMU-69620 | Cell Cycle Checkpoints | 5 | 265 | 0.0252 | Psmb4,Ppp2r1a,Ppp2ca,Ywhaz,Psma3 |
| MMU-1640170 | Cell Cycle | 7 | 574 | 0.0261 | Akt1,Psmb4,Ppp2r1a,Ppp2ca,Ywhaz,Hsp90aa1,Psma3 |
| MMU-187037 | Signaling by NTRK1 (TRKA) | 3 | 64 | 0.0261 | Map2k1,Ppp2r1a,Ppp2ca |
| MMU-450604 | KSRP (KHSRP) binds and destabilizes mRNA | 2 | 14 | 0.0269 | Akt1,Ywhaz |
| MMU-5358565 | Mismatch repair (MMR) directed by MSH2:MSH6 (MutSalpha) | 2 | 14 | 0.0269 | Msh6,Msh2 |
| MMU-8948751 | Regulation of PTEN stability and activity | 3 | 68 | 0.0269 | Akt1,Psmb4,Psma3 |
| MMU-9006934 | Signaling by Receptor Tyrosine Kinases | 6 | 418 | 0.0269 | Akt1,Map2k1,Ctnnb1,Ppp2r1a,Ppp2ca,Hsp90aa1 |
| MMU-69202 | Cyclin E associated events during G1/S transition | 3 | 70 | 0.0275 | Akt1,Psmb4,Psma3 |
| MMU-1295596 | Spry regulation of FGF signaling | 2 | 16 | 0.0289 | Ppp2r1a,Ppp2ca |
| MMU-180024 | DARPP-32 events | 2 | 16 | 0.0289 | Ppp2r1a,Ppp2ca |
| MMU-3700989 | Transcriptional Regulation by TP53 | 5 | 296 | 0.0289 | Akt1,Ppp2r1a,Ppp2ca,Ywhaz,Lamtor4 |
| MMU-432142 | Platelet sensitization by LDL | 2 | 16 | 0.0289 | Ppp2r1a,Ppp2ca |
| MMU-5628897 | TP53 Regulates Metabolic Genes | 3 | 72 | 0.0289 | Akt1,Ywhaz,Lamtor4 |
| MMU-69656 | Cyclin A:Cdk2-associated | 3 | 72 | 0.0289 | Akt1,Psmb4,Psma3 |

| | | | | | |
|---|---|---|---|---|---|
| | events at S phase entry | | | | |
| MMU-8852276 | The role of GTSE1 in G2/M progression after G2 checkpoint | 3 | 73 | 0.0289 | Psmb4,Hsp90aa1,Psma3 |
| MMU-2995383 | Initiation of Nuclear Envelope (NE) Reformation | 2 | 18 | 0.0315 | Ppp2r1a,Ppp2ca |
| MMU-8878159 | Transcriptional regulation by RUNX3 | 3 | 81 | 0.0338 | Psmb4,Ctnnb1,Psma3 |
| MMU-2467813 | Separation of Sister Chromatids | 4 | 184 | 0.0351 | Psmb4,Ppp2r1a,Ppp2ca,Psma3 |
| MMU-69278 | Cell Cycle, Mitotic | 6 | 490 | 0.0419 | Akt1,Psmb4,Ppp2r1a,Ppp2ca,Hsp90aa1,Psma3 |
| MMU-69273 | Cyclin A/B1/B2 associated events during G2/M transition | 2 | 24 | 0.0479 | Ppp2r1a,Ppp2ca |
| WP373 | IL-3 signaling pathway | 5 | 98 | 0.0014 | Akt1,Map2k1,Bcl2l1,Ppp2ca,Ywhaz |
| WP387 | IL-6 signaling pathway | 5 | 98 | 0.0014 | Akt1,Map2k1,Ppp2r1a,Ppp2ca,Hsp90aa1 |
| WP1763 | PluriNetWork: mechanisms associated with pluripotency | 6 | 287 | 0.0070 | Akt1,Map2k1,Ctnnb1,Ppp2r1a,Trim33,Klf4 |
| WP1257 | Mismatch repair | 2 | 9 | 0.0175 | Msh6,Msh2 |
| WP151 | IL-5 signaling pathway | 3 | 67 | 0.0312 | Akt1,Ctnnb1,Ywhaz |
| WP450 | IL-2 signaling pathway | 3 | 75 | 0.0356 | Akt1,Map2k1,Hsp90aa1 |
| WP1560 | MicroRNAs in cardiomyocyte hypertrophy | 3 | 82 | 0.0368 | Akt1,Map2k1,Ctnnb1 |
| WP246 | TNF-alpha NF-kB signaling pathway | 4 | 180 | 0.0368 | Akt1,Ppp2ca,Ywhaz,Hsp90aa1 |
| WP2841 | Focal adhesion: PI3K-Akt-mTOR signaling pathway | 5 | 318 | 0.0368 | Akt1,Map2k1,Ppp2r1a,Ppp2ca,Hsp90aa1 |

| | | | | | |
|---|---|---|---|---|---|
| WP10 | IL-9 signaling pathway | 2 | 23 | 0.0374 | Akt1,Map2k1 |
| WP1249 | EPO receptor signaling | 2 | 26 | 0.0374 | Akt1,Map2k1 |
| WP662 | Amino acid metabolism | 3 | 95 | 0.0374 | Suclg1,Ldha,Aldh7a1 |
| WP723 | Wnt signaling pathway and pluripotency | 3 | 96 | 0.0374 | Ctnnb1,Ppp2r1a,Ppp2ca |
| WP539 | Wnt signaling pathway (NetPath) | 3 | 106 | 0.0398 | Akt1,Ctnnb1,Ppp2ca |
| WP317 | Glycogen metabolism | 2 | 32 | 0.0459 | Ppp2r1a,Ppp2ca |
| WP339 | ESC pluripotency pathways | 3 | 116 | 0.0459 | Akt1,Map2k1,Ctnnb1 |
| MP:0010768 | Mortality/aging | 33 | 5549 | 0.00093 | S100a6,Akt1,Map2k1,Msh6,Napa,Ctnnb1,Ppp2r1a,Bcl2l1,Nutf2,Ppp2ca,Mtdh,Ywhaz,Prkdc,Msh2,Trim33,Sh2b1,Fmnl1,Slc7a1,Eif5a,Lamtor4,Suclg1,Hsp90aa1,Pigt,Klf4,Ldha,Pnpla6,Hecw2,Aktip,Ccbe1,Tecr,Slc3a2,Fam96b,Aldh7a1 |
| MP:0010769 | Abnormal survival | 32 | 5150 | 0.00093 | S100a6,Akt1,Map2k1,Msh6,Napa,Ctnnb1,Ppp2r1a,Bcl2l1,Nutf2,Ppp2ca,Ywhaz,Prkdc,Msh2,Trim33,Sh2b1,Fmnl1,Slc7a1,Eif5a,Lamtor4,Suclg1,Hsp90aa1,Pigt,Klf4,Ldha,Pnpla6,Hecw2,Aktip,Ccbe1,Tecr,Slc3a2,Fam96b,Aldh7a1 |
| MP:0010770 | Preweaning lethality | 30 | 4663 | 0.00093 | S100a6,Akt1,Map2k1,Napa,Ctnnb1,Ppp2r1a,Bcl2l1,Nutf2,Ppp2ca,Ywhaz,Prkdc,Trim33,Sh2b1,Fmnl1,Slc7a1,Eif5a,Lamtor4,Suclg1,Hsp90aa1,Pigt,Klf4,Ldha,Pnpla6,Hecw2,Aktip,Ccbe1,Tecr,Slc3a2,Fam96b, Aldh7a1 |
| MP:0011182 | Decreased hematopoietic cell number | 13 | 1172 | 0.0165 | Ctnnb1,Bcl2l1,Ppp2ca,Prkdc,Msh2,Fmnl1,Slc7a1,Afmid,Klf4,Ldha,Myo1c,Ccbe1,Slc3a2 |
| BTO:0000379 | Embryo | 18 | 1528 | 8.92e-05 | S100a6,Akt1,Ctnnb1,Ppp2r1a,Mtdh,Ywhaz,Msh2,Trim33,Eif5a,Suclg1,Hsp90aa1,Klf4,Ldha,Myo1c,Ccbe1,Psma3,Tecr,Slc3a2 |
| BTO:0000421 | Connective tissue | 19 | 1868 | 0.00017 | S100a6,Akt1,Ctnnb1,Ppp2r1a,Pcbd2,Mtdh,Trim33,Fmnl1,Slc7a1,Eif5a,Suclg1,Hsp90aa1,Klf4,Ldha,Myo1c,Pnpla6,Psma3,Tecr,Slc3a2 |
| BTO:0000284 | Organism form | 19 | 1948 | 0.00021 | S100a6,Akt1,Ctnnb1,Ppp2r1a,Mtdh,Ywhaz,Msh2,Trim33,Sh2b1,Eif5a,Suclg1,Hsp90aa1,Klf4,Ldha,Myo1c,Ccbe1,Psma3,Tecr,Slc3a2 |
| BTO:0004669 | Finger | 4 | 28 | 0.00074 | Akt1,Ctnnb1,Hsp90aa1,Klf4 |
| BTO:0000452 | Fibroblast | 9 | 494 | 0.0018 | S100a6,Ppp2r1a,Trim33,Slc7a1,Suclg1,Hsp90aa1,Klf4,Ldha,Myo1c |
| BTO:0000000 | Tissues, cell types and enzyme sources | 40 | 9281 | 0.0053 | S100a6,Akt1,Map2k1,Msh6,Psmb4,Napa,Ctnnb1,Ppp2r1a,Bcl2l1,Syngr1,Pcbd2,Rab2b,Mtdh,Ywhaz,Msh2,Trim33,Sh2b1,Cyp2s1,Fmnl1,Slc7a1,Eif5a,Lamtor4,Suclg1,Cpsf6,Afmid,Hsp90aa1,Pigt,Atp5d,Klf4, Ldha,Myo1c,Pnpla6,Rad51ap1,Wdr45,Ccbe1,Psma3,Tecr,Slc3a2,Fam96b,Aldh7a1 |
| BTO:0000759 | Liver | 14 | 1546 | 0.0069 | Akt1,Msh6,Psmb4,Ctnnb1,Mtdh,Sh2b1,Suclg1,Afmid,Hsp90aa1,Atp5d,Ldha,Psma3,Tecr,Aldh7a1 |
| BTO:0000775 | Lymphocyte | 6 | 220 | 0.0069 | Akt1,Map2k1,Ctnnb1,Bcl2l1,Fmnl1,Hsp90aa1 |
| BTO:0002572 | MEF cell | 3 | 20 | 0.0069 | Akt1,Ctnnb1,Hsp90aa1 |
| BTO:0001489 | Whole body | 39 | 9158 | 0.0075 | S100a6,Akt1,Map2k1,Msh6,Psmb4,Napa,Ctnnb1,Ppp2r1a,Bcl2l1,Syngr1,Pcbd2,Rab2b,Mtdh,Ywhaz,Msh2,Trim33,Sh2b1,Cyp2s1,Fmnl1,Slc7a1,Eif5a,Lamtor4,Suclg1,Cpsf6,Afmid,Hsp90aa1,Pigt,Atp5d,Klf4, Ldha,Myo1c,Pnpla6,Rad51ap1,Wdr45,Ccbe1,Psma3,Tecr,Slc3a2,Aldh7a1 |
| BTO:0004253 | Plant candle | 2 | 3 | 0.0111 | Psmb4,Psma3 |
| BTO:0000944 | NIH-3T3 cell | 3 | 28 | 0.0112 | Akt1,Ctnnb1,Hsp90aa1 |
| BTO:0000091 | Ascites | 3 | 31 | 0.0140 | Akt1,Ctnnb1,Hsp90aa1 |
| BTO:0000391 | DLD-1 cell | 2 | 4 | 0.0140 | Akt1,Ctnnb1 |

| | | | | | |
|---|---|---|---|---|---|
| BTO:0000970 | Osteosarcoma cell | 2 | 4 | 0.0140 | Akt1,Ctnnb1 |
| BTO:0001415 | Umbilical cord | 3 | 31 | 0.0140 | Akt1,Ctnnb1,Klf4 |
| BTO:0001461 | Whole plant | 6 | 282 | 0.0140 | Akt1,Psmb4,Ctnnb1,Klf4,Psma3,Fam96b |
| BTO:0000345 | Digestive gland | 15 | 2031 | 0.0169 | Akt1,Msh6,Psmb4,Ctnnb1,Mtdh,Sh2b1,Slc7a1,Suclg1,Afmid,Hsp90aa1,Atp5d,Ldha,Psma3,Tecr,Aldh7a1 |
| BTO:0000498 | Gastric cancer cell | 2 | 6 | 0.0191 | Akt1,Ctnnb1 |
| BTO:0001129 | Prostate gland | 3 | 41 | 0.0203 | Akt1,Ctnnb1,Hsp90aa1 |
| BTO:0000089 | Blood | 7 | 467 | 0.0205 | Akt1,Map2k1,Ctnnb1,Bcl2l1,Fmnl1,Hsp90aa1,Klf4 |
| BTO:0001491 | Viscus | 22 | 3991 | 0.0216 | S100a6,Akt1,Msh6,Psmb4,Napa,Ctnnb1,Mtdh,Trim33,Sh2b1,Fmnl1,Slc7a1,Suclg1,Cpsf6,Afmid,Hsp90aa1,Atp5d,Klf4,Ldha,Pnpla6,Psma3,Tecr,Aldh7a1 |
| BTO:0000182 | HT-29 cell | 2 | 8 | 0.0265 | Akt1,Ctnnb1 |
| BTO:0000256 | Myoblast cell line | 3 | 49 | 0.0281 | Akt1,Ctnnb1,Hsp90aa1 |
| BTO:0001243 | Shoot | 4 | 127 | 0.0294 | Akt1,Psmb4,Psma3,Fam96b |
| BTO:0001615 | Colorectal cancer cell | 2 | 10 | 0.0332 | Akt1,Ctnnb1 |
| BTO:0000123 | Bladder | 3 | 56 | 0.0363 | Akt1,Ctnnb1,Mtdh |
| BTO:0000047 | Adrenal gland | 3 | 60 | 0.0398 | Akt1,Ctnnb1,Hsp90aa1 |
| BTO:0000269 | Colon | 4 | 143 | 0.0398 | S100a6,Akt1,Msh6,Ctnnb1 |
| BTO:0000294 | Dermis | 3 | 59 | 0.0398 | Akt1,Ctnnb1,Klf4 |
| BTO:0000586 | Colonic cancer cell | 2 | 12 | 0.0398 | Akt1,Ctnnb1 |
| BTO:0000669 | Embryonic cell line | 4 | 147 | 0.0407 | Akt1,Ctnnb1,Hsp90aa1,Klf4 |
| BTO:0000753 | Lymphoid tissue | 13 | 1858 | 0.0461 | Akt1,Map2k1,Napa,Ctnnb1,Bcl2l1,Trim33,Fmnl1,Slc7a1,Lamtor4,Cpsf6,Hsp90aa1,Klf4,Rad51ap1 |
| BTO:0000782 | T-lymphocyte | 4 | 157 | 0.0461 | Akt1,Map2k1,Ctnnb1,Hsp90aa1 |
| BTO:0001078 | Placenta | 4 | 155 | 0.0461 | Akt1,Ctnnb1,Klf4,Myo1c |
| BTO:0001967 | Cervical cancer cell line | 2 | 14 | 0.0461 | Akt1,Ywhaz |
| BTO:0000608 | Hepatoma cell | 2 | 15 | 0.0466 | Akt1,Ctnnb1 |
| GOCC:0005622 | Intracellular | 46 | 9943 | 8.61e-05 | S100a6,Akt1,Map2k1,Msh6,Psmb4,Napa,Ctnnb1,Ppp2r1a,Bcl2l1,Nutf2,Syngr1,Glod4,Ppp2ca,Pcbd2,Mtdh,Ywhaz,Prkdc,Pdia5,Msh2,Arl8a,Trim33,Sh2b1,Brat1,Cyp2s1,Cog5,Fmnl1,Eif5a,Lamtor4,Suclg1,Cpsf6,Afmid,Hsp90aa1,Scrt1,Pigt,Atp5d,Klf4,Ldha,Myo1c,Pnpla6,Rad51ap1,Wdr45,Hecw2,Aktip,Psma3,Fam96b,Aldh7a1 |
| GOCC:0005737 | Cytoplasm | 36 | 6579 | 0.00023 | S100a6,Akt1,Map2k1,Psmb4,Napa,Ctnnb1,Ppp2r1a,Bcl2l1,Syngr1,Glod4,Ppp2ca,Mtdh,Ywhaz,Pdia5,Arl8a,Sh2b1,Brat1,Cyp2s1,Cog5,Fmnl1,Eif5a,Lamtor4,Suclg1,Afmid,Hsp90aa1,Pigt,Atp5d,Klf4,Ldha,Myo1c,Pnpla6,Wdr45,Aktip,Psma3,Fam96b,Aldh7a1 |
| GOCC:0032991 | Protein-containing complex | 29 | 4654 | 0.00045 | S100a6,Akt1,Msh6,Psmb4,Napa,Ctnnb1,Ppp2r1a,Bcl2l1,Nutf2,Ppp2ca,Prkdc,Msh2,Cog5,Eif5a,Lamtor4,Suclg1,Cpsf6,Hsp90aa1,Pigt,Atp5d,Klf4,Ldha,Myo1c,Creg1,Aktip,Psma3,Slc3a2,Fam96b,Aldh7a1 |
| GOCC:0110165 | Cellular anatomical entity | 49 | 12108 | 0.00045 | S100a6,Akt1,Map2k1,Msh6,Psmb4,Napa,Ctnnb1,Ppp2r1a,Bcl2l1,Nutf2,Syngr1,Glod4,Ppp2ca,Mtdh,Ywhaz,Prkdc,Pdia5,Msh2,Arl8a,Trim33,Sh2b1,Brat1,Cyp2s1,Cog5,Fmnl1,Slc7a1,Eif5a,Lamtor4,Suclg1,Cpsf6,Afmid,Hsp90aa1,Scrt1,Pigt,Atp5d,Klf4,Ldha,Myo1c,Pnpla6,Creg1,Rad51ap1,Wdr45,Aktip,Ccbe1,Psma3,Tecr,Slc3a2,Fam96b,Aldh7a1 |
| GOCC:0005829 | Cytosol | 16 | 1784 | 0.0033 | S100a6,Akt1,Map2k1,Psmb4,Ctnnb1,Ppp2r1a,Bcl2l1,Ppp2ca,Ywhaz,Sh2b1,Fmnl1,Hsp90aa1,Ldha,Myo1c,Psma3,Fam96b |
| GOCC:0043227 | Membrane-bounded organelle | 35 | 7176 | 0.0033 | S100a6,Akt1,Map2k1,Msh6,Ctnnb1,Bcl2l1,Nutf2,Syngr1,Glod4,Mtdh,Ywhaz,Prkdc,Pdia5,Msh2,Trim33,Brat1,Cyp2s1,Cog5,Fmnl1,Eif5a,Lamtor4,Suclg1,Cpsf6,Hsp90aa1,Scrt1,Pigt,Atp5d,Klf4,Ldha,Myo1c,Pnpla6,Rad51ap1,Tecr,Fam96b,Aldh7a1 |
| GOCC:0043229 | Intracellular organelle | 37 | 8073 | 0.0043 | S100a6,Akt1,Map2k1,Msh6,Ctnnb1,Ppp2r1a,Bcl2l1,Nutf2,Syngr1,Glod4,Ppp2ca,Mtdh,Ywhaz,Prkdc,Pdia5,Msh2,Arl8a,Trim33,Brat1,Cyp2s1,Cog5,Fmnl1,Eif5a,Lamtor4,Suclg1,Cpsf6,Hsp90aa1,Scrt1,Pigt,Atp5d,Klf4,Ldha,Myo1c,Pnpla6,Rad51ap1,Fam96b,Aldh7a1 |
| GOCC:0043226 | Organelle | 38 | 8565 | 0.0055 | S100a6,Akt1,Map2k1,Msh6,Ctnnb1,Ppp2r1a,Bcl2l1,Nutf2,Syngr1,Glod4,Ppp2ca,Mtdh,Ywhaz,Prkdc,Pdia5,Msh2,Arl8a,Trim33,Brat1,Cyp2s1,Cog5,Fmnl1,Eif5a,Lamtor4,Suclg1,Cpsf6,Hsp90aa1,Scrt1,Pigt,Atp5d,Klf4,Ldha,Myo1c,Pnpla6,Rad51ap1,Tecr,Fam96b,Aldh7a1 |

| | | | | | |
|---|---|---|---|---|---|
| GOCC:0043231 | Intracellular membrane-bounded organelle | 31 | 6320 | 0.0102 | S100a6,Akt1,Map2k1,Msh6,Ctnnb1,Bcl2l1,Nutf2,Glod4,Mtdh,Ywhaz,Prkdc,Pdia5,Msh2,Trim33,Brat1,Cyp2s1,Cog5,Eif5a,Lamtor4,Suclg1,Cpsf6,Hsp90aa1,Scrt1,Pigt,Atp5d,Klf4,Ldha,Pnpla6,Rad51ap1,Fam96b,Aldh7a1 |
| GOCC:0032302 | MutSbeta complex | 2 | 4 | 0.0211 | Msh6,Msh2 |
| GOCC:0032301 | MutSalpha complex | 2 | 5 | 0.0268 | Msh6,Msh2 |
| GOCC:1902494 | Catalytic complex | 12 | 1413 | 0.0417 | Akt1,Msh6,Psmb4,Ppp2r1a,Ppp2ca,Prkdc,Suclg1,Pigt,Ldha,Psma3,Fam96b,Aldh7a1 |
| GOCC:1990391 | DNA repair complex | 3 | 44 | 0.0417 | Msh6,Prkdc,Msh2 |
| KW-0007 | Acetylation | 24 | 3070 | 0.00011 | S100a6,Akt1,Msh6,Psmb4,Napa,Ctnnb1,Ppp2r1a,Nutf2,Syngr1,Pcbd2,Mtdh,Ywhaz,Prkdc,Msh2,Trim33,Eif5a,Lamtor4,Suclg1,Hsp90aa1,Atp5d,Ldha,Myo1c,Psma3,Aldh7a1 |
| KW-0963 | Cytoplasm | 28 | 4763 | 0.0012 | S100a6,Akt1,Map2k1,Psmb4,Ctnnb1,Ppp2r1a,Bcl2l1,Nutf2,Ppp2ca,Mtdh,Ywhaz,Arl8a,Sh2b1,Brat1,Cog5,Fmnl1,Eif5a,Cpsf6,Afmid,Hsp90aa1,Ldha,Myo1c,Wdr45,Hecw2,Aktip,Psma3,Fam96b,Aldh7a1 |
| KW-0539 | Nucleus | 27 | 4635 | 0.0016 | S100a6,Akt1,Map2k1,Msh6,Psmb4,Ctnnb1,Ppp2r1a,Bcl2l1,Nutf2,Ppp2ca,Mtdh,Prkdc,Msh2,Trim33,Sh2b1,Brat1,Eif5a,Cpsf6,Afmid,Hsp90aa1,Scrt1,Klf4,Myo1c,Rad51ap1,Psma3,Fam96b,Aldh7a1 |
| KW-0653 | Protein transport | 8 | 586 | 0.0183 | Napa,Nutf2,Rab2b,Arl8a,Cog5,Eif5a,Myo1c,Aktip |
| KW-0159 | Chromosome partition | 3 | 45 | 0.0265 | Ppp2r1a,Arl8a,Fam96b |
| KW-0906 | Nuclear pore complex | 3 | 44 | 0.0265 | Nutf2,Eif5a,Myo1c |
| KW-0702 | S-nitrosylation | 3 | 53 | 0.0318 | Ctnnb1,Prkdc,Hsp90aa1 |

**Supplementary table 2.** Most significant biological process

| Term ID | Term description | Observed gene count | Background gene count | False discovery rate | Matching proteins in your network (labels) |
|---|---|---|---|---|---|
| **GO:0043231** | Intracellular membrane-bounded organelle | 46 | 9507 | 1.17e-05 | S100a6,Akt1,Map2k1,Msh6,Psmb4,Napa,Ctnnb1,Ppp2r1a,Bcl2l1,Nutf2,Glod4,Ppp2ca,Pcbd2,Rab2b,Mtdh,Ywhaz,Prkdc,Pdia5,Msh2,Arl8a,Cyb5r1,Trim33,Sh2b1,Brat1,Cyp2s1,Cog5,Eif5a,Lamtor4,Suclg1,Cpsf6,Afmid,Hsp90aa1,Scrt1,Pigt,Atp5d,Klf4,Ldha,Myo1c,Pnpla6,Rad51ap1,Aktip,Psma3 Tecr,Slc3a2,Fam96b,Aldh7a1 |
| **GO:0051052** | Regulation of DNA metabolic process | 9 | 365 | 0.0058 | Akt1,Msh6,Ctnnb1,Prkdc,Msh2,Sh2b1,Hsp90aa1,Klf4,Rad51ap1 |
| **GO:0007346** | Regulation of mitotic cell cycle | 9 | 531 | 0.0197 | Akt1,Map2k1,Ctnnb1,Bcl2l1,Prkdc,Msh2,Sh2b1,Klf4,Hecw2 |
| **GO:0009987** | Cellular process | 49 | 13330 | 0.0302 | S100a6,Akt1,Map2k1,Msh6,Psmb4,Napa,Ctnnb1,Ppp2r1a,Bcl2l1,Nutf2,Syngr1,Ppp2ca,Pcbd2,Rab2b,Mtdh,Ywhaz,Prkdc,Pdia5,Msh2,Arl8a,Trim33,Sh2b1,Brat1,Cyp2s1,Cog5,Fmnl1,Slc7a1,Eif5a,Lamtor4,Suclg1,Cpsf6,Afmid,Hsp90aa1,Scrt1,Pigt,Atp5d,Klf4,Ldha,Myo1c,Pnpla6,Rad51ap1,<br><br>Wdr45, Hecw2Aktip,Psma3,Tecr,Slc3a2,Fam96b,Aldh7a1 |
| **GO:0006915** | Apoptotic process | 10 | 825 | 0.0457 | Akt1,Msh6,Bcl2l1,Prkdc,Msh2,Brat1,Eif5a,Hsp90aa1,Pigt,Aktip |
| **GO:0008630** | Intrinsic apoptotic signaling pathway in response to DNA damage | 4 | 75 | 0.0457 | Msh6,Bcl2l1,Prkdc,Msh2 |
| **GO:0044237** | Cellular metabolic process | 31 | 6445 | 0.0457 | Akt1,Map2k1,Msh6,Psmb4,Ctnnb1,Ppp2r1a,Ppp2ca,Pcbd2,Ywhaz,Prkdc,Msh2,Trim33,Cyp2s1,Eif5a,Suclg1,Cpsf6,Afmid,Hsp90aa1,Pigt,Atp5d,Klf4,Ldha,Pnpla6,Rad51ap1,Wdr45,Hecw2,Aktip,Psma3,Tecr, Fam96b,Aldh7a1 |
| **GO:0051726** | Regulation of cell cycle | 11 | 1016 | 0.0457 | Akt1,Map2k1,Ctnnb1,Ppp2r1a,Bcl2l1,Ppp2ca,Prkdc,Msh2,Sh2b1,Klf4,Hecw2 |
| **GO:0055114** | Oxidation-reduction process | 10 | 917 | 0.0479 | Akt1,Pcbd2,Pdia5,Cyb5r1,Cyp2s1,Suclg1,Atp5d,Ldha,Tecr,Aldh7a1 |
| **GO:1903827** | Regulation of cellular protein localization | 8 | 576 | 0.0479 | Akt1,Map2k1,Ctnnb1,Bcl2l1,Nutf2,Ppp2ca,Hsp90aa1,Myo1c |
| **GO:0006886** | Intracellular protein transport | 9 | 759 | 0.0488 | Akt1,Napa,Nutf2,Syngr1,Rab2b,Ywhaz,Eif5a,Hsp90aa1,Myo1c |

| | | | | | |
|---|---|---|---|---|---|
| **GO:0010564** | Regulation of cell cycle process | 8 | 583 | 0.0488 | Akt1,Ctnnb1,Ppp2r1a,Bcl2l1,Prkdc,Sh2b1,Klf4,Hecw2 |
| **GO:0019899** | Enzyme binding | 19 | 2277 | 0.0070 | Akt1,Map2k1,Msh6,Ctnnb1,Bcl2l1,Nutf2,Ppp2ca,Ywhaz,Prkdc,Msh2,Sh2b1,Fmnl1,Lamtor4,Hsp90aa1,Klf4,Myo1c,Wdr45,Ccbe1,Psma3 |
| **GO:0044877** | Protein-containing complex binding | 15 | 1411 | 0.0070 | Map2k1,Msh6,Napa,Ctnnb1,Bcl2l1,Ppp2ca,Ywhaz,Msh2,Fmnl1,Eif5a,Suclg1,Cpsf6,Atp5d,Myo1c,Ccbe1 |
| **GO:0019901** | Protein kinase binding | 10 | 707 | 0.0142 | Akt1,Map2k1,Ctnnb1,Bcl2l1,Ppp2ca,Ywhaz,Msh2,Sh2b1,Hsp90aa1,Wdr45 |

**Supplementary table 3.** Overrepresented pathway

| #category | Pathway ID | Term description | Observed gene count | Background gene count | False discovery rate | Matching proteins in your network (labels) |
|---|---|---|---|---|---|---|
| UniProt Keywords | KW-0007 | Acetylation | 24 | 3070 | 0.00011 | S100a6,Akt1,Msh6,Psmb4,Napa,Ctnnb1,Ppp2r1a,Nutf2,Syngr1,Pcbd2,Mtdh,Ywhaz,Prkdc,Msh2,Trim33,Eif5a,Lamtor4,Suclg1,Hsp90aa1,Atp5d,Ldha,Myo1c,Psma3,Aldh7a1 |
| TISSUES | BTO:0000421 | Connective tissue | 19 | 1868 | 0.00017 | S100a6,Akt1,Ctnnb1,Ppp2r1a,Pcbd2,Mtdh,Trim33,Fmnl1,Slc7a1,Eif5a,Suclg1,Hsp90aa1,Klf4,Ldha,Myo1c,Pnpla6,Psma3,Tecr,Slc3a2 |
| Reactome | MMU-168256 | Immune System | 15 | 1621 | 0.0040 | Akt1,Psmb4,Ctnnb1,Ppp2r1a,Bcl2l1,Syngr1,Ppp2ca,Ywhaz,Arl8a,Sh2b1,Hsp90aa1,Myo1c,Creg1,Hecw2,Psma3 |
| TISSUES | BTO:0000452 | Fibroblast | 9 | 494 | 0.0018 | S100a6,Ppp2r1a,Trim33,Slc7a1,Suclg1,Hsp90aa1,Klf4,Ldha,Myo1c |
| KEGG | mmu04151 | PI3K-Akt signaling pathway | 7 | 355 | 0.0032 | Akt1,Map2k1,Ppp2r1a,Bcl2l1,Ppp2ca,Ywhaz,Hsp90aa1 |
| Reactome | MMU-1257604 | PIP3 activates AKT signaling | 6 | 234 | 0.0061 | Akt1,Psmb4,Ppp2r1a,Ppp2ca,Lamtor4,Psma3 |
| WikiPathways | WP373 | IL-3 signaling pathway | 5 | 98 | 0.0014 | Akt1,Map2k1,Bcl2l1,Ppp2ca,Ywhaz |
| WikiPathways | WP387 | IL-6 signaling pathway | 5 | 98 | 0.0014 | Akt1,Map2k1,Ppp2r1a,Ppp2ca,Hsp90aa1 |
| WikiPathways | WP2841 | Focal adhesion: PI3K-Akt-mTOR signaling pathway | 5 | 318 | 0.0368 | Akt1,Map2k1,Ppp2r1a,Ppp2ca,Hsp90aa1 |
| WikiPathways | WP246 | TNF-alpha NF-kB signaling pathway | 4 | 180 | 0.0368 | Akt1,Ppp2ca,Ywhaz,Hsp90aa1 |
| KEGG | mmu04210 | Apoptosis | 3 | 135 | 0.0489 | Akt1,Map2k1,Bcl2l1 |
| WikiPathways | WP151 | IL-5 signaling pathway | 3 | 67 | 0.0312 | Akt1,Ctnnb1,Ywhaz |
| WikiPathways | WP450 | IL-2 signaling pathway | 3 | 75 | 0.0356 | Akt1,Map2k1,Hsp90aa1 |
